# Resilient modulus characteristics and model prediction of cement phosphogypsum under dry-wet cycling conditions

**DOI:** 10.1371/journal.pone.0341539

**Published:** 2026-09-08

**Authors:** Wengang Gan, Kaisheng Chen, Jinxiong Chen, Yu’ang Chen, Shuang Deng

**Affiliations:** 1 College of Civil Engineering, Guizhou University, Guiyang, China; 2 School of Civil Engineering, Southwest Jiaotong University, Chengdu, China; 3 School of Architectural Engineering, Kaili University, Kaili, Guizhou, China; University of Queensland, AUSTRALIA

## Abstract

The subgrade resilient modulus is a crucial parameter in pavement structure design, directly related to the safety and economy of the pavement structure. Currently, the subgrade resilient modulus used in pavement structure design is the static resilient modulus. To address this issue, this paper employs dynamic triaxial tests to analyze the effects of confining stress ($\sigma_{\text{3}}$), cyclic dynamic stress ($\sigma_{\text{d}}$), wet-dry cycles (N), and cement dosage (C) on the resilient modulus (M_R_). It explores the relationship among the M_R_, static resilient modulus, and 7-day unconfined compressive strength ($\sigma_{c}$), and proposes a predictive model for M_R_ considering C and N. SEM and XRD methods are used to analyze the mechanism of the effect of wet-dry cycles on M_R_ at the microstructural level. The results show that; (1) The M_R_ of the PG mixture increases with the rise of $\sigma_{\text{3}}$ and decreases with the increase of $\sigma_{\text{d}}$, is positively correlated with C, and negatively correlated with the number of N, with the decay of M_R_ mainly concentrated in the first four wet-dry cycles and gradually stabilizing afterwards, which is explained through SEM and XRD analysis. (2) By comparing M_R_ and the static resilient modulus, it is found that the M_R_ of PG specimens under wet-dry cycles is 1.25–1.30 times that of the static resilient modulus, this ratio can be used to make a preliminary estimation of the M_R_ of C-stabilized PG materials. (3) The model relating $\sigma_{c}$ and M_R_ follows a linear relationship, with correlation coefficients for the fit all exceeding 0.94, indicating a good fit. (4) A model for predicting M_R_ that incorporates N and C was proposed, and the reliability of this model was verified by comparing predicted values with experimental data. The predicted M_R_ provides a reliable basis for pavement design and holds significant engineering value.

## 1. Introduction

PG is a solid waste derivative produced in the wet-process phosphoric acid industry [1]. Statistics show that five tons of PG are generated for every ton of phosphoric acid produced [2]. With the increasing agricultural demand and continuous growth of the phosphate fertilizer industry, the production of PG shows an upward trend, resulting in a large accumulation of PG. Taking Guizhou as an example, the output of PG in Guizhou Province was about 18.5 million tons in 2024, with a historical stockpile as high as 200 million tons. PG contains trace amounts of silicon, fluorides, phosphorus pentoxide, organic compounds, rare earth elements, and radioactive substances [3–6]. For the environment, the large amount of fluorides in PG can contaminate groundwater and surface water. Such polluted water is also rich in phosphorus, which is prone to cause water eutrophication and damage aquatic ecosystems. Long-term intake of high-fluoride water by humans can not only cause dental fluorosis but also lead to bone deformation and skeletal fluorosis. In addition, since PG contains a large amount of heavy metals and radioactive substances, inhalation of PG dust or consumption of contaminated water can damage human liver and kidney functions as well as the nervous system. It is also accompanied by risks such as anemia and carcinogenicity. Therefore, the development and utilization of PG has become a research focus and has attracted extensive attention from many scholars. At present, PG is applied in construction, agriculture, chemical industry and other fields through various approaches [6]. In the construction industry, PG can be used to manufacture innovative bricks and cement-based materials [7,8]. In the chemical industry, it is mainly used to enhance the properties of cementitious materials and produce cement-based subgrade composites. In agriculture, PG is used as a soil stabilization material to prepare alkaline soil conditioners [9,10]. PG can also be used in the field of filling, serving as a basic aggregate to restore stability in mining areas, promote resource recycling, and reduce the risk of geological disasters. The comprehensive utilization of PG allows its application as a new type of phase change material [11], uranium adsorption material, and porous sound-absorbing material [12,13].

Besides the uses of phosphate gypsum (PG) mentioned above, many researchers have been exploring the feasibility of using PG in highway engineering. Using PG in road base materials can not only help absorb industrial by-products on a large scale and ease the pressure of open-air PG stacking, but also partially replace natural sand and gravel aggregates, reducing the mining of mineral resources and energy consumption, cutting construction costs, and supporting the development of a circular economy. This brings notable economic and social benefits. C-PG stabilized macadam materials were prepared by partially replacing fine aggregates with PG, and the influencing factors of water stability and crack resistance were analyzed, and the feasibility of the mix ratio was verified through experiments [14]. Combined with the special engineering properties of PG and red clay, cement (lime) was used as a curing agent to establish a model to reflect the dynamic strength index of the mixture [15]. The mechanical properties and durability of the new PG material were studied. By measuring indexes such as unconfined compressive strength, splitting strength, dry-wet cycles and freeze-thaw cycles, it was verified that the mixture meets the design standards for base materials of highways and first-class highways [16]. Liu et al. used PG as a base course material for sub-base paving [17,18], and Lu G et al. used PG as a surface course material to produce artificial aggregates and asphalt modifiers [19,20]. PG used as subgrade material is also regarded as the optimal choice for large PG consumption with low pavement safety risks [21,22]. However, the application market still faces challenges such as limited application fields, low market acceptance, low utilization rate, high production cost and regional restrictions.

Resilient modulus (M_R_) is a key parameter for evaluating subgrade stiffness and characterizing the deformation resistance of soil under repeated traffic loads. Since M_R_ can quantitatively reflect the mechanical properties of subgrade materials, it has become a focus in geotechnical engineering research. Numerous studies have discussed the effects of various internal and external factors on M_R_ through laboratory tests. Among internal factors, moisture content has consistently been identified as the primary variable; many studies show that M_R_ decreases with increasing moisture content, highlighting the moisture sensitivity of subgrade materials [23–25] Zhang et al. further demonstrated that the degree of compaction and matrix suction exert a significant influence on M_R_ [26]. Similarly, other researchers have emphasized the influence of initial dry density and particle size distribution, which modulate soil structure and thereby regulate its elastic response [27,28]. The results of Nazerke S et al. show that M_R_ exhibits a decreasing trend as the number of freeze-thaw cycles increases, with the rate of M_R_ decline slowing as the freeze-thaw process progresses [29,30].

Based on the aforementioned research, the effect of PG as a subgrade material on M_R_ under different N and C conditions remains to be investigated. Therefore, this study examines the M_R_ of PG under heavy traffic conditions in regions with seasonal rainfall, with particular attention to its material properties and mechanical evolution. This study utilized dynamic triaxial tests to analyze the effects of confining stress ($\sigma_{\text{3}}$), cyclic dynamic stress $\left(\sigma_{\text{d}}\right)$, the number of wet-dry cycles (N) and cement dosage (C) on the resilient modulus (M_R_). The relationship between M_R_ and the static resilient modulus, as well as the 7-day unconfined compressive strength ($\sigma_{\text{c}}$), was examined. A predictive model for M_R_ that accounts for C and N was proposed, and SEM and XRD methods were employed to analyze the microstructure and elucidate the mechanism by which wet-dry cycles affect M_R_. This research fills a knowledge gap regarding the influence of C and N on M_R_ and holds significant engineering value.

## 2. Materials and methods

### 2.1. Materials

**Fig 1:**
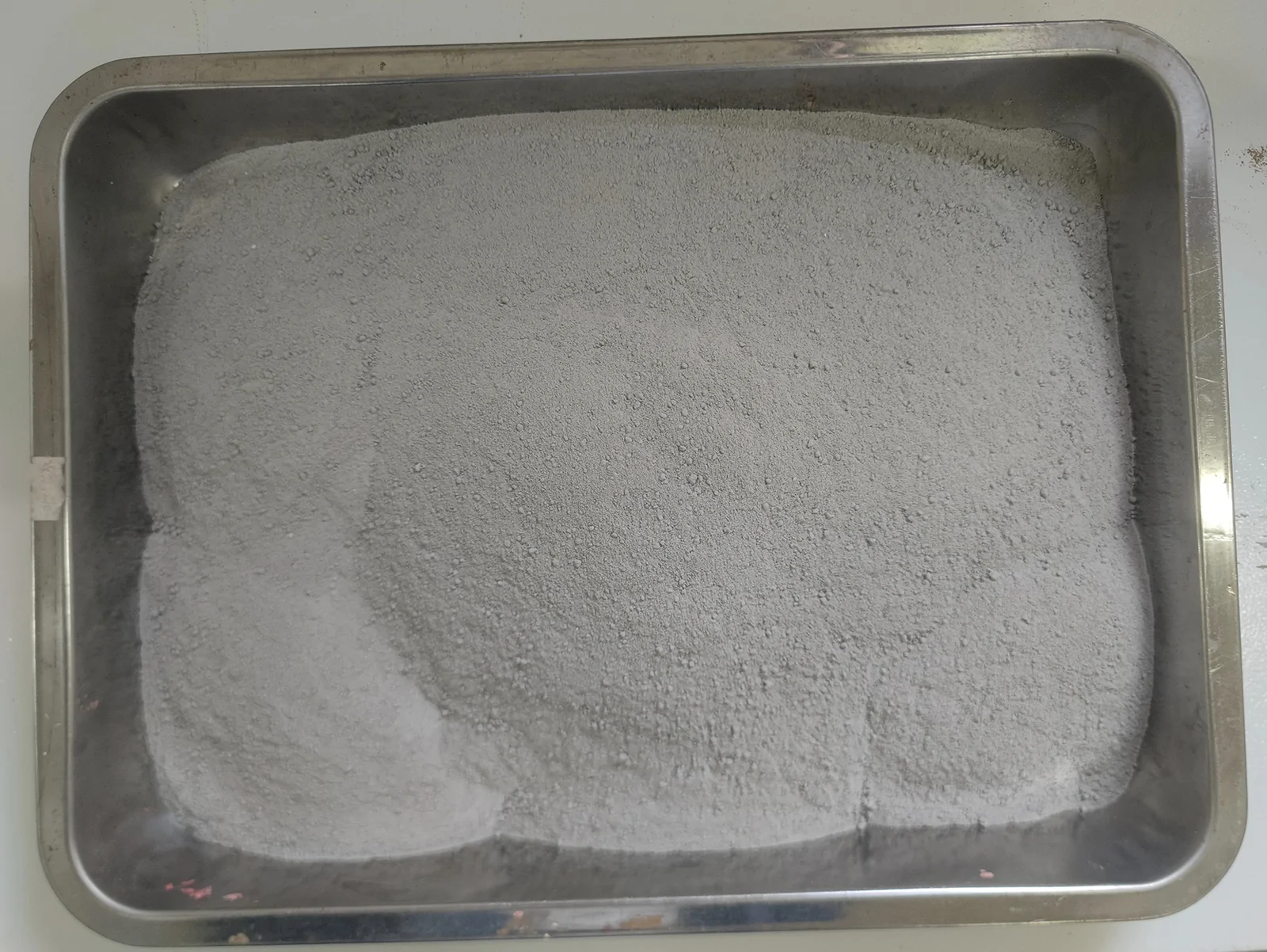
PG used in the test.

**Fig 2:**
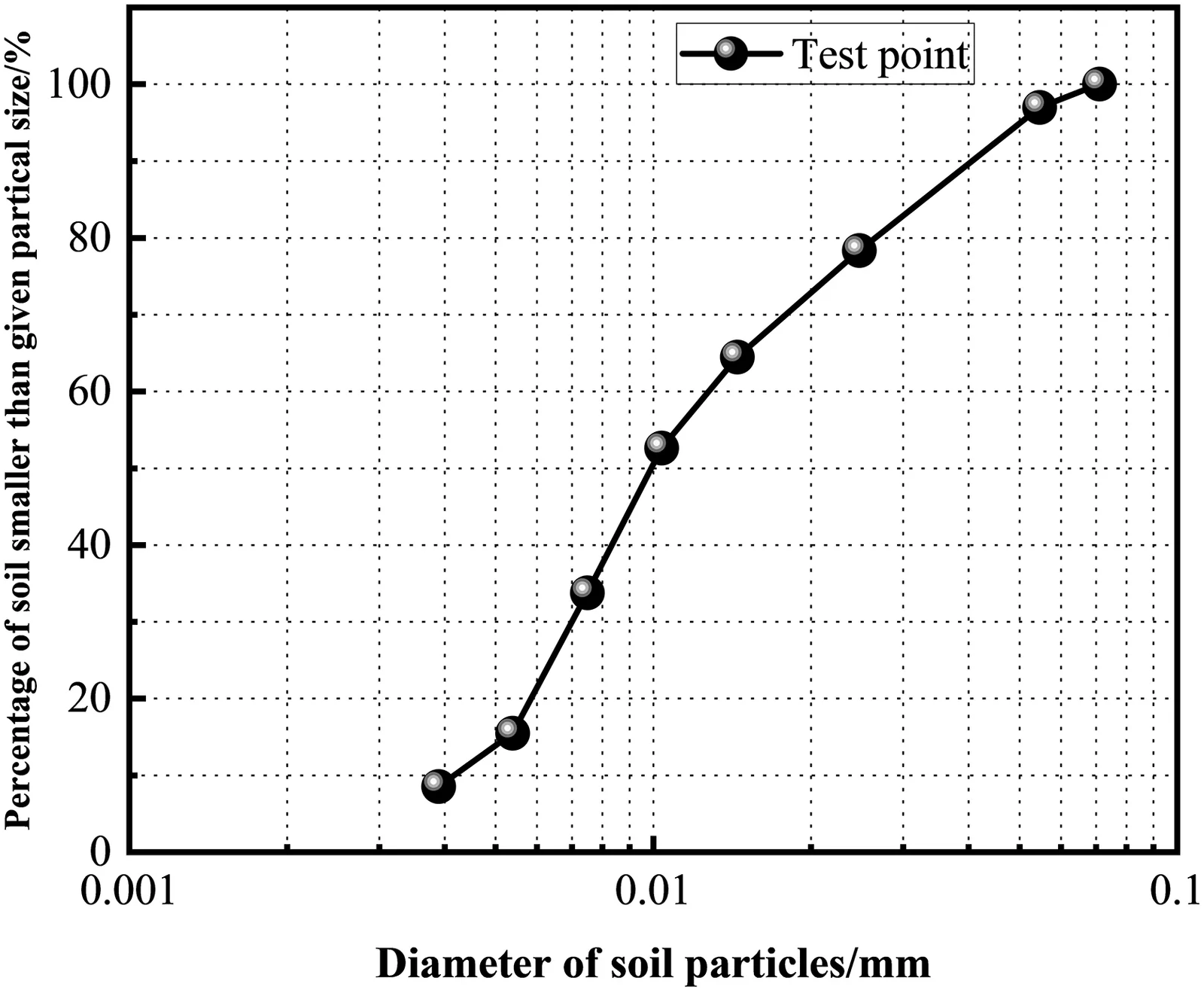
PG particle grading curve.

The PG used in this experiment was obtained from Wengfu Phosphate Mine in Qiannan, Guizhou Province. It appears as an off-white powder in a dry state, as shown in Fig 1. Since PG is a fine-grained soil, a hydrometer method was adopted for supplementary sieve analysis to obtain more accurate particle gradation characteristics. A Type-A hydrometer was used in the test, and the PG particle gradation curve was plotted based on the results, as shown in Fig 2. The characteristic particle sizes of the PG sample are as follows: D_60_ (control particle size) = 0.0144, D_30_ (median particle size) = 0.0074, D_10_ (effective particle size) = 0.0048, C_U_ (coefficient of uniformity) = D_60_ × D_10_^−1^ = 0.0144/0.0048 = 3.00, C_C_ (coefficient of curvature) = D_30_ × D_30_ × D_60_^^−1^^ × D_10_^^−1^^ = 0.00774 × 0.0074 × 0.0144^^−1^^ × 0.0048^^−1^^ = 0.79, C_U_ = 3.00 is less than 5, C_C_ = 0.79, the PG gradation is uniform but poorly graded. The proportion of PG particles <0.075mm is 57.65%, exceeding 50%, making it fine-grained soil.

The main components of the raw material are CaO, SO_3_ and SiO_2_, and its main chemical compositions are listed in Table 1. The basic physical indexes of PG are shown in Table 2. Since PG contains potentially harmful substances like phosphorus and fluorine, it should be tested for heavy metal content and radioactivity before the experiment. The test results are shown in Tables 3 and 4. The cement used in the experiment is P·C 32.5R Portland cement of a brand from Southwest China, which is dry and free of agglomeration. The basic parameters and chemical compositions are presented in Tables 5 and 6. Tests show that all indexes of the cement satisfy the technical specifications of Common Portland Cement (GB 175–2007).

**Table 1:** Chemical Composition of PG.

Ingredient	SO_3_	CaO	SiO_2_	P_2_O_5_	Na_2_O	Al_2_O_3_	Other
Mass fraction/(%)	50.44	39.26	6.05	1.47	0.49	0.55	1.74

**Table 2:** Basic physical parameters of PG.

Wet density /(g ∙ cm^-3^)	Dry density /(g ∙ cm^-3^)	Water content/(%)	Liquid limit ($w_{l}$)/(%)	Plastic limit ($w_{p}$)/(%)	Plasticity index ($I_{p}$)/(%)
1.23	1.07	14.5	27.5	13.7	13.8

**Table 3:** PG radioactivity test results.

Testing items	Technical requirements:	Test results	Single decision	Detection conclusion
Cra (Bq/kg)	/	53.89	/	Complies with the requirements of the national standard GB 6566−2010 “Limits for Radionuclides in Building Materials”
CTh (Bq/kg)	/	42.09	/	
CK(Bq/kg)	/	53.89	/	
Internal irradiation index(Ira)	≤1.0	0.4	Qualified	
External beam index (Ir)	≤1.0	0.4	Qualified	

**Table 4:** Test results of hazardous components in PG leachate.

Testing items	Technical requirements:	Test results	Single decision	Detection conclusion
Cu(mg/L)	100	0.157	Qualified	Meets the requirements of GB 5085.3−2007 “Identification of Hazardous Waste Identification Standards for Leaching Toxicity”
Zn(mg/L)	100	0.051	Qualified	
Cd(mg/L)	1	–	Qualified	
Pb(mg/L)	5	–	Qualified	
Cr(mg/L)	15	–	Qualified	
As(mg/L)	5	0.0361	Qualified	
Hg(mg/L)	0.1	0.0006	Qualified	

**Table 5:** Basic parameters of cement.

setting time/(min)		$f_{cf}$/(MPa)		$f_{cu}$/(MPa)	
Initial	Final	3/(d)	28/(d)	3/(d)	28/(d)
302	322	5.9	6.7	24.9	43.7

**Table 6:** Chemical Composition of PG of cement.

Ingredient	CaO	SiO_2_	Al_2_O_3_	Fe_2_O_3_	MgO	Other
Mass fraction/(%)	63.5	22.4	4.5	3.3	2.2	4.1

### 2.2. Mixture proportion design and sample preparation

**Fig 3:**
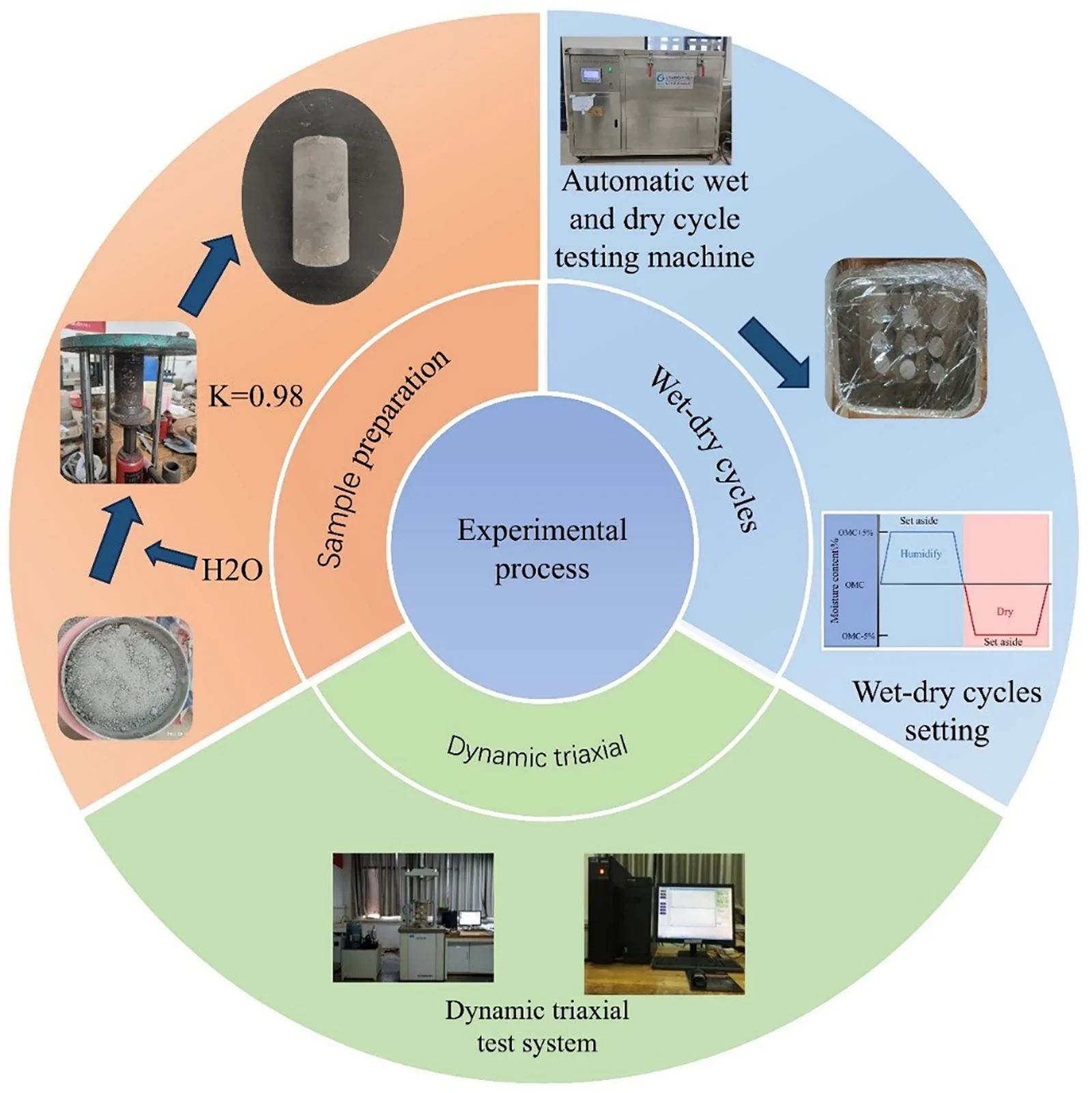
Flowchart of the testing process.

The overall experimental procedure is shown in Fig 3 below.

#### 2.2.1. Mixture proportion design.

**Fig 4:**
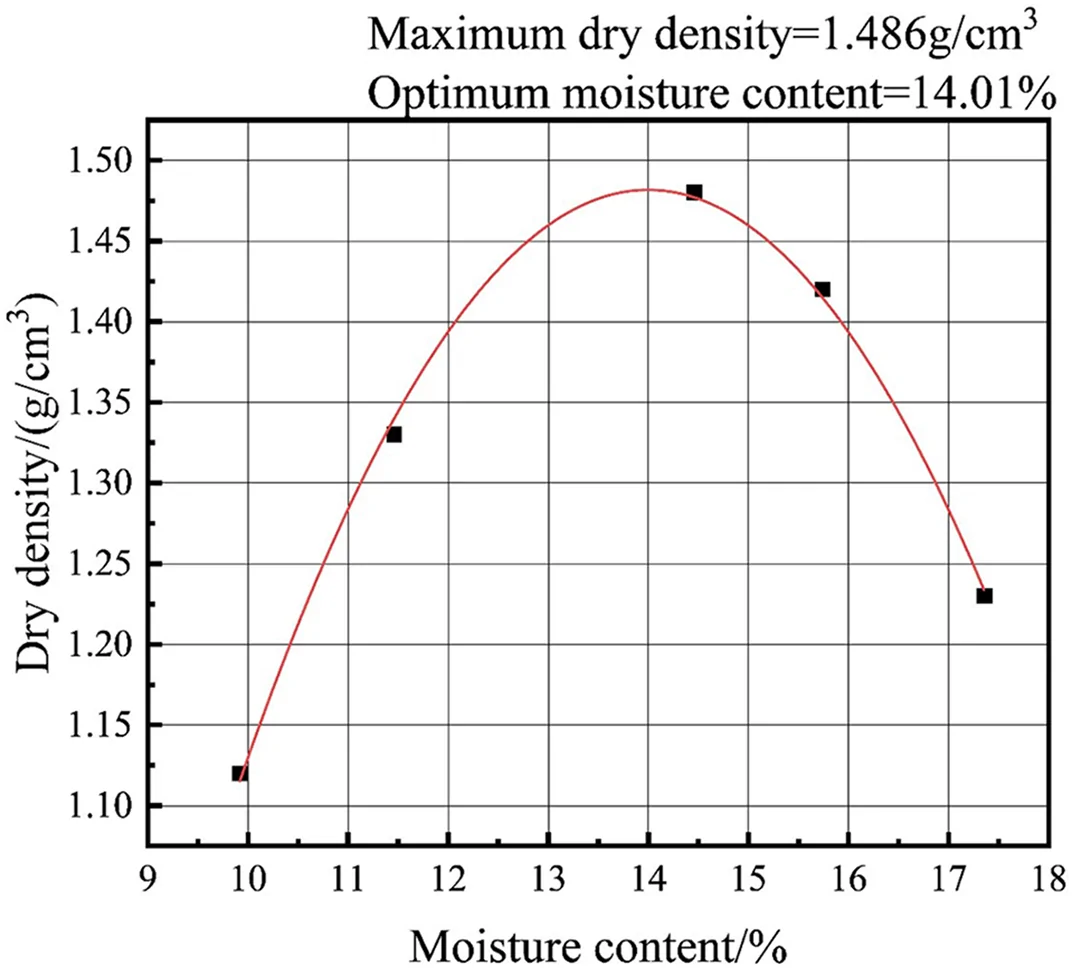
Water content and dry density curve under C = 9%.

**Fig 5:**
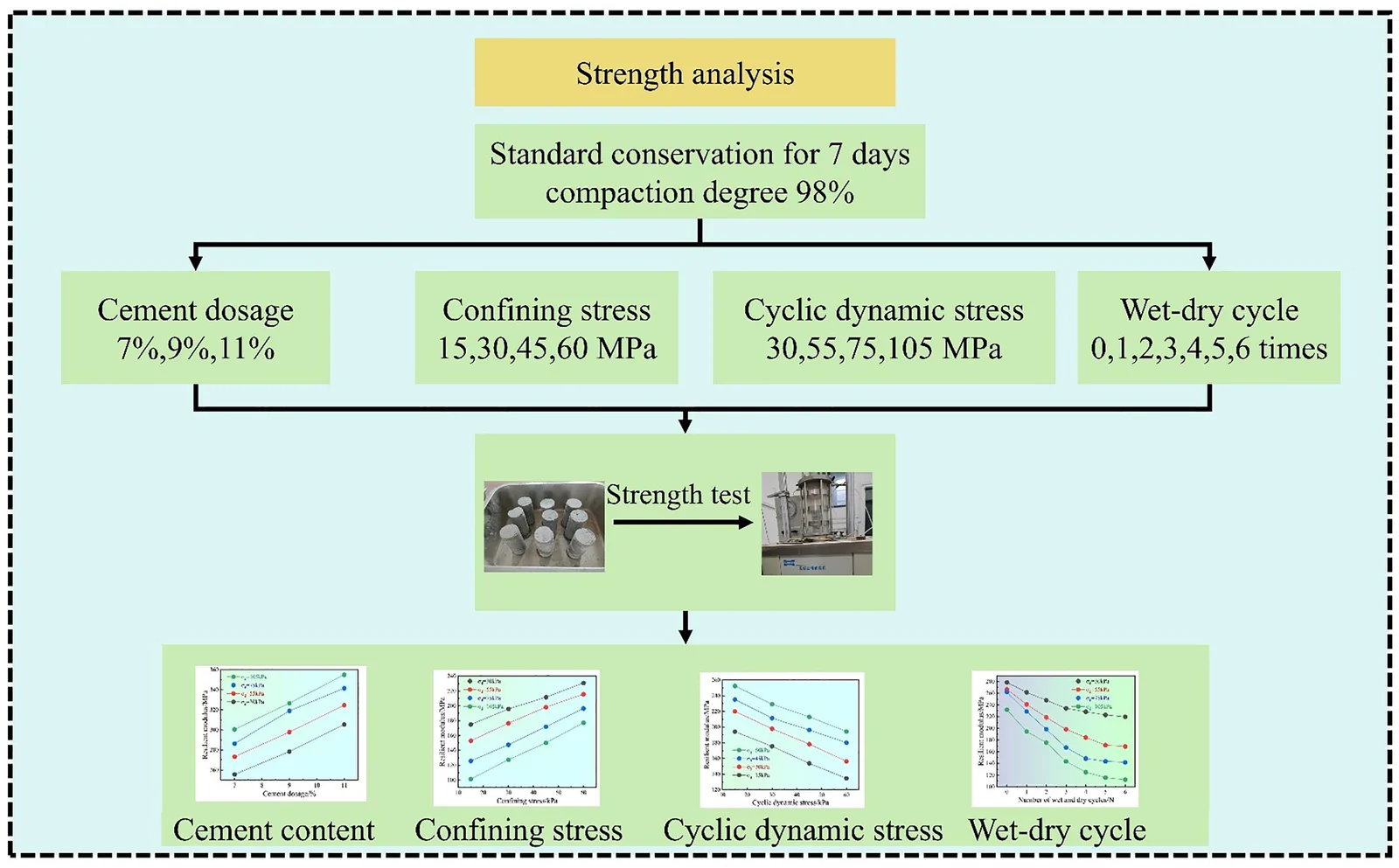
Sample preparation scheme and strength test.

Extensive studies have been conducted on the mix proportion of C-PG materials worldwide. According to the Technical Specifications for Construction of Highway Pavement Base Courses (JTG/T F20-2015), when cement-stabilized soil is used as the subbase and the plasticity index of the soil is greater than 12, the recommended cement dosage is 6%–14%. Scholars such as Mansheng Dong et al. conducted in-depth research on cement-stabilized macadam bases and found that $\sigma_{\text{c}}$ of the mixture reaches the maximum when the cement dosage is 5% and the C:PG ratio is 1:3, which can meet the strength requirements of pavement bases [31,32]. Mingkai Zhou et al. carried out unconfined compressive strength tests on C-PG-stabilized macadam and found that the compressive strength peaks at a cement dosage of 5% [33]. Zhengfu Qian used 10% cement to modify PG, and unconfined compressive strength tests showed that C-PG-stabilized materials improved by the curing agent exhibit good long-term strength performance [34]. Based on previous research, C-PG-stabilized materials with cement dosage of 7%, 9%, and 11% can meet the specification requirements for pavement bases and subbases in most cases. Therefore, these three cement dosage levels were adopted for sample preparation in this experiment. The cement dosage was calculated in accordance with Technical Specifications for Construction of Highway Pavement Base Courses (JTG/T F20-2015), expressed as the percentage of cement mass to the total mass of dry stabilized materials. Compaction tests were then performed on the mixture following the Standard for Highway Geotechnical Tests (JTG 3430−2020). The compaction results are shown in Table 7. The compaction results at 9% cement dosage are presented in Fig 4. Specimens were prepared according to the compaction test results. All samples were statically compacted at the optimum moisture content with a compaction degree of 98%. The specimens were cylindrical with a height of 80 mm and a diameter of 39.1 mm. The sample preparation scheme and strength tests are shown in Fig 5.

**Table 7:** C-PG mixture compaction test results.

Cement dosage/(%)	Compaction/(%)	Maximum Dry Density/(g ∙ cm^-3^)	Optimum Moisture Content/(%)
7	98	1.515	14.56
9		1.486	14.01
11		1.502	14.66

#### 2.2.2. Preparation of samples.

**Fig 6:**
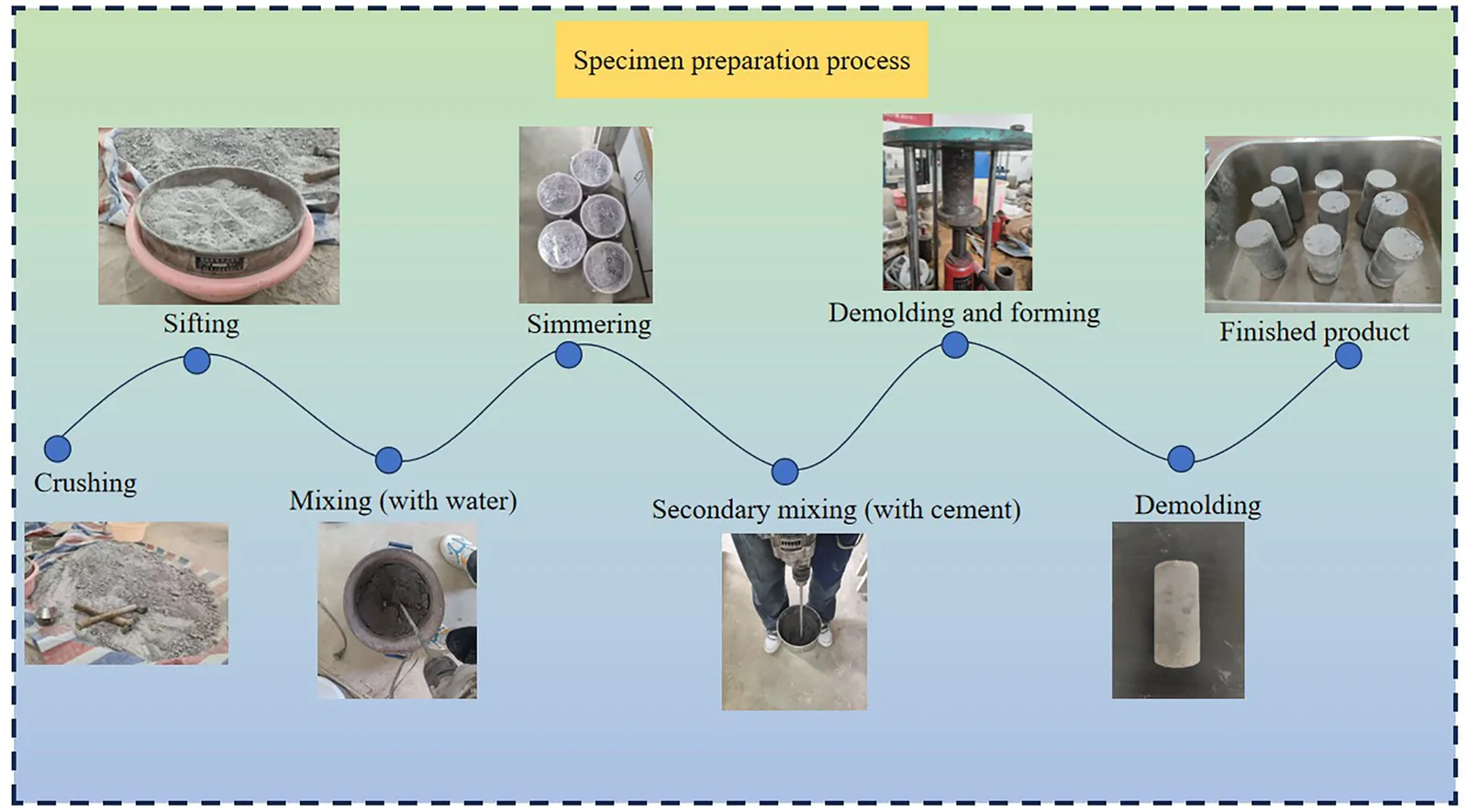
Specimen preparation process.

The sample preparation method follows the static pressure method specified in the Test Procedures for Inorganic Binder Stabilized Materials in Highway Engineering (JTG_3441–2024) [35]. Before specimen preparation, the PG used in the test was placed in an oven at 40°C and dried for 24 hours to evaporate most of its internal moisture. The moisture content before and after must be measured by weighing, and it must be less than 1% to be used for the experiment. The bulk PG was crushed with a hammer or other tools and then sieved through a 2 mm sieve. Subsequently, the first mixing was conducted. According to the designed compaction degree and optimum moisture content, the required masses of PG and water were calculated. All the weighed PG and 90% of the required water were poured into a bucket and mixed uniformly with a hand-held mixer. The well-mixed PG was sealed and stored for 24 hours to allow sufficient water absorption. Then the second mixing was performed. After 24 hours of standing, the PG was mixed with the remaining 10% of the required water, and the corresponding mass of cement was added simultaneously, followed by thorough mixing again with the mixer. The mixture after the second mixing was poured into the mold and manually compacted and demolded using a hydraulic jack. The duration from the end of the second mixing to the completion of specimen preparation should not exceed 1 hour, since excessive time may lead to premature hardening of cement and thus affect the properties of the specimens. Finally, standard curing was carried out. The prepared specimens were placed in a pre-prepared tray with permeable stones underneath, sealed with plastic wrap, and then placed in a curing chamber at a temperature of 18–22°C and a humidity of no less than 95%. The specimen preparation process is shown in Fig 6.

### 2.3. Test methods

#### 2.3.1. Wet-dry cycle program.

When PG is used as subgrade filling material, it will inevitably be affected by factors such as groundwater level rise, rainfall infiltration, and solar radiation under open-air conditions. The material will undergo dry-wet cycles. Under repeated dry-wet cycles, the internal structure of the subgrade will be damaged, eventually leading to problems such as reduced subgrade strength and subgrade cracking, thus resulting in potential safety hazards. Harianto Rahardjo et al. utilized analyses of slope stability based on the temporal variations in pore water pressure and soil moisture during wet and dry periods [36]. When calculating the shear strength of unsaturated soils using water-soil characteristic curves, Qian et al. took into account the inherent uncertainty of these curves and proposed correcting the SWCC values obtained from conventional Tempé cells or pressure plates by considering different stress levels, in order to assess the shear strength of unsaturated soils [37]. Ye et al. converted sampled soil data into continuous field-scale outputs of unsaturated soil properties for incorporation into slope stability analyses [38]. Therefore, studying the mechanism and influence law of moisture content of road materials on subgrade performance is of great significance for optimizing subgrade design and ensuring engineering safety. To simulate real field conditions as closely as possible, this paper simplifies the influence of material moisture content on roads through dry-wet cycles. Studies have shown that the dry-wet cycle path has a significant impact on the strength of base materials. In general, the strength degradation caused by wetting first and then drying is much greater than that caused by drying first and then wetting. Therefore, the wetting-first and drying-later path was adopted in this study [39,40].The amplitude of dry-wet cycles (the upper limit moisture content for wetting and the lower limit moisture content for drying) also affects the strength of the mixture to a certain extent. According to the research results in references [41,42], After the sub-base material has been compacted at its optimal moisture content, exposure to natural climatic conditions for a period of time will cause the moisture content to increase, fluctuating periodically or non-periodically within a range of ±5% of the ‘optimal moisture content’ (OMC). To simulate this real-world engineering scenario, and taking into account the liquid and plastic limit moisture contents of PG, a moisture variation range of 10% has been established; that is, during the wetting process, the moisture content of the test specimen is adjusted to 5% above the optimal moisture content, and during the drying process, the moisture content of the specimen is reduced to 5% below the initial moisture content. The main steps of a complete wet-dry cycle test are as follows: (1) Wetting stage: Remove the plastic wrap from the cured specimens and place them in a curing box. Turn on the humidifier to wet the specimens by spraying. Take out the specimens at intervals and monitor their moisture content by weighing. When the moisture content reaches the upper limit (OMC + 5%), stop wetting and seal the specimens for 24 hours. To ensure uniform moisture distribution inside the specimens, a pre-experiment was conducted in this study. The specimens were first placed in a dry-wet cycle test chamber for cycles, and parts of the specimens from the upper, middle and lower layers were taken to measure the moisture content by the drying method. Uniform moisture distribution was confirmed when the difference in moisture content among the three layers was less than 1%. The formal test was started only after calibrating the instrument. (2) Drying stage: The wetted specimens were dried in an oven at 40°C. Similarly, the moisture content of the specimens was measured by weighing at intervals. When the moisture content reached the drying target (OMC – 5%), the specimens were taken out and drying was stopped. After standing for 24 hours, the specimens were rewetted to the optimum moisture content, completing one dry-wet cycle. Subsequent cycles were performed in the same manner.

#### 2.3.2. Dynamic triaxial test.

The resilient modulus test was carried out using an SDT-20 microcomputer-controlled dynamic triaxial test machine for soil. According to the Standard for Highway Geotechnical Tests (JTG 3430−2020), the stress state experienced by different subgrade regions under vehicle load is governed by the coupling effect of $\sigma_{\text{3}}\;$and $\sigma_{d}$ [43]. Given the strong stress dependence of the subgrade soil M_R_ [44], the applied test conditions must be as close as possible to the in-situ stress environment. The selection of triaxial test parameters was guided by empirical observations and field data. Specifically, $\sigma_{\text{3}}\;$was set to 15, 30, 45, and 60 kPa, which are consistent with the ranges commonly specified in various pavement layers. Field investigations have shown that $\sigma_{\text{3}}$ in subgrade soils typically spans 0–60 kPa [45]. To account for heavy traffic effects, the load response at different depths within the subgrade was examined. The results indicate that the amplitude of $\sigma_{d}$ can reach 150 kPa at a depth of 0.5 m below the subgrade surface, while a smaller $\sigma_{d}$ is still observed at 3.5 m depth [46]. Therefore, $\sigma_{d}$ values of 30, 55, 75, and 105 kPa were adopted to reflect a gradient of stress conditions, including those aggravated by traffic growth and related stress amplification effects.

During the test, the confining stress supply line and the triaxial cell were connected first. The standard loading sequence for fine-grained soil specimens is listed in Table 8. A pre-loading confining stress of 30 kPa was applied to the specimen, where $\sigma_{\text{max}}=\sigma_{c\text{1}}+\sigma_{\text{d}}$. A haversine pulse load was used as the test load, and at least 1000 cycles of haversine pulse load were applied to the specimen at a frequency of 10 Hz. The loading duration and rest period were 0.1 s and 0.9 s, respectively. The maximum axial stress of the specimen could be taken as 61.0 kPa. For each loading sequence, after applying the corresponding number of pulse loads, the dynamic resilient deformations of the last 5 cycles in the sequence were selected. If these 5 deformation values showed no obvious fluctuation and tended to be constant, the specimen was judged to have reached a stable elastic deformation response. The M_R_ under that sequence was calculated using the average value of these 5 deformations. The formula for calculating the M_R_ is given in Equation (1).

**Table 8:** Loading sequence of fine-grained soil specimens.

Load the serial number	confining stress $\sigma_{3}$/(Kpa)	Contact stress $\sigma_{c1}$/(Kpa)	cyclic dynamic stress $\sigma_{d}$/(Kpa)	axial stress $\sigma_{max}$/(Kpa)	The number of loads acted
0	30	6	55	61	1000
1	60	12	30	42	100
2	45	9	30	39	100
3	30	6	30	36	100
4	15	3	30	33	100
5	60	12	55	67	100
6	45	9	55	64	100
7	30	6	55	61	100
8	15	3	55	58	100
9	60	12	75	87	100
10	45	9	75	84	100
11	30	6	75	81	100
12	15	3	75	78	100
13	60	12	105	117	100
14	45	9	105	114	100
15	30	6	105	111	100
16	15	3	105	108	100


$$\begin{array}{c} {\text{M}}_{\text{R}}=\frac{\sigma_{\text{d}}}{\epsilon_{\text{R}}} \end{array}$$ (1)

In the formula: M_R_ denotes the resilient modulus (MPa); $\sigma_{d}$ denotes the cyclic dynamic stress (MPa); $\epsilon_{\text{R}}$ denotes the axial resilient strain.

#### 2.3.3. Unconfined compressive strength test.

Place the properly cured specimens onto the unconfined compressive strength testing machine for compression testing. In accordance with the “Test Procedures for Inorganic Binder Stabilized Materials in Highway Engineering” (JTG3441−2024), maintain a loading rate of 1 mm/min throughout the test. Record the maximum pressure at specimen failure during testing. Then calculate the compressive strength using the unconfined compressive strength formula (2).


$$\begin{array}{c} R_{C}=\frac{P}{A} \end{array}$$ (2)

In the formula: R_C_ denotes the unconfined compressive strength of the specimen (MPa); P denotes the maximum pressure at specimen failure (kN); A denotes the cross-sectional area of the specimen (mm²).

#### 2.3.4. Microtesting program.

SEM and XRD tests were conducted to analyze the strength development mechanism under different dry-wet cycles from the perspectives of particle morphology, arrangement characteristics (pores) and chemical composition of the specimens. The influence of each factor could be obtained by comparing the micro results of different groups. The mixture was prepared by the static pressure method and taken out after standard curing. Then the specimens were immersed in anhydrous ethanol to stop the hydration reaction. After that, they were placed in an oven and dried at 40°C to remove free water until the weight remained constant. The specimens were taken out, crushed and processed into particles with a length and width of 1–2 cm (generally not less than 1 cm) and a thickness of no more than 10 mm. The particles were placed in a German ZEISS GeminiSEM 300 scanning electron microscope to observe the morphology characteristics of the specimen section. In addition, a part of the crushed specimens was ground into powder with a particle size of about 0.05 mm for X-ray diffraction test to analyze the composition under different conditions.

## 3. Results and discussion

### 3.1. Effects of confining stress on resilient modulus

**Fig 7:**
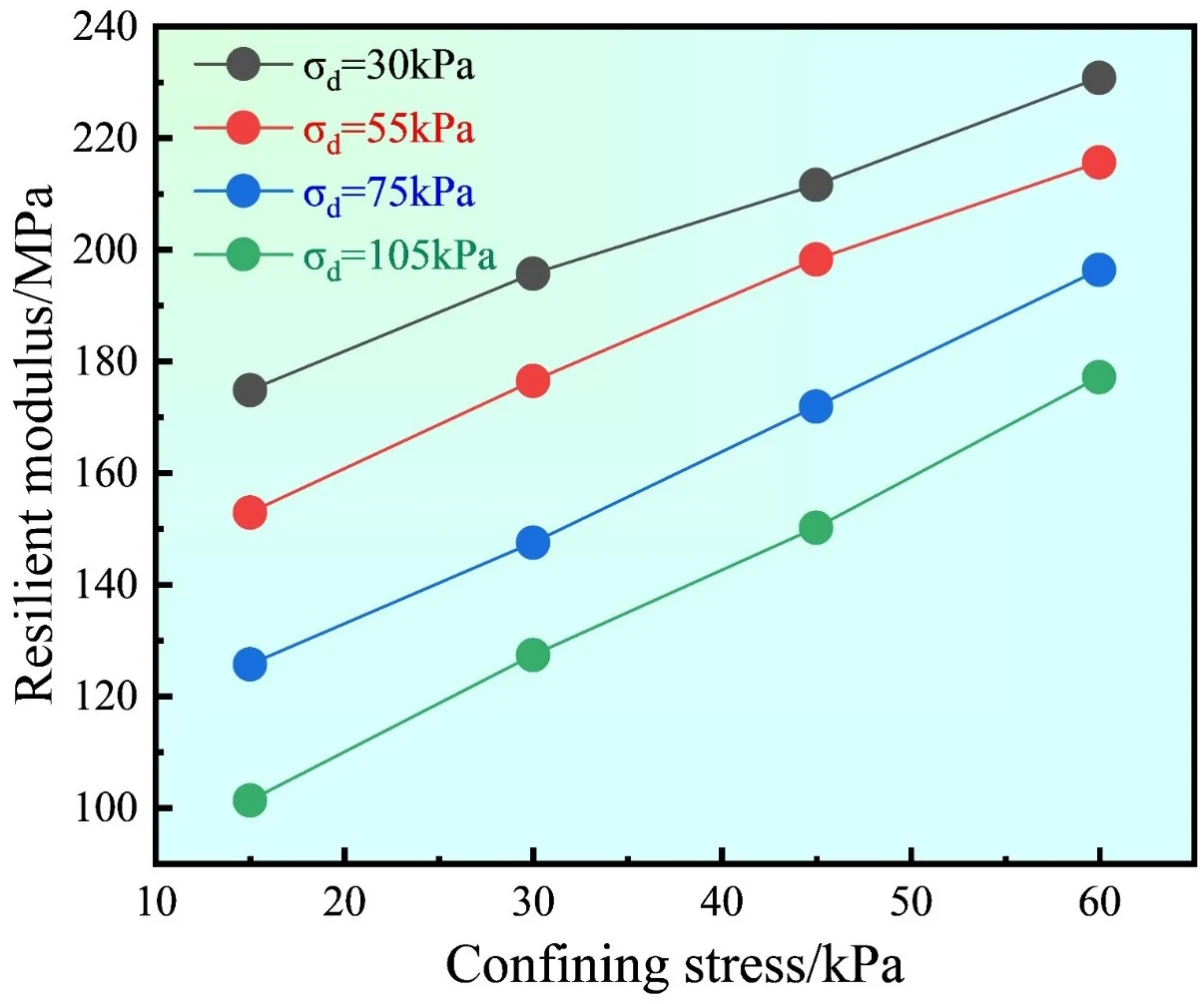
The relationship between M_R_ and $\sigma_{3}\;$with different $\sigma_{𝐝}$.

Fig 7 shows the relationship between M_R_ and $\sigma_{\text{3}}\;$for specimens tested at C = 7% and N = 6. similar patterns are observed for other wet-dry cycles, due to space constraints, these are not shown in detail here. The figure indicates that, for a given $\sigma_{\text{d}}$. The M_R_ of the test specimen is positively correlated with $\sigma_{\text{3}}$ and the relationship is approximately linear. Taking $\sigma_{\text{d}}$=55 kPa as an example, when $\sigma_{\text{3}}\;$is 15 kPa, 30 kPa, 45 kPa and 60 kPa, the corresponding M_R_ values are 152.84 MPa, 176.48 MPa, 198.24 MPa and 215.64 MPa, representing increases of 12.47%, 12.33% and 8.76% respectively. Analyzing the underlying causes, the presence of confining stress subjects the specimen to lateral pressure, restricting its lateral deformation during loading. This enhances the interlocking and friction between the particles within the specimen, reduces its porosity, and thereby improves its resistance to deformation and failure, consequently increasing its strength.

### 3.2. Effects of Cyclic dynamic Stress on resilient modulus

**Fig 8:**
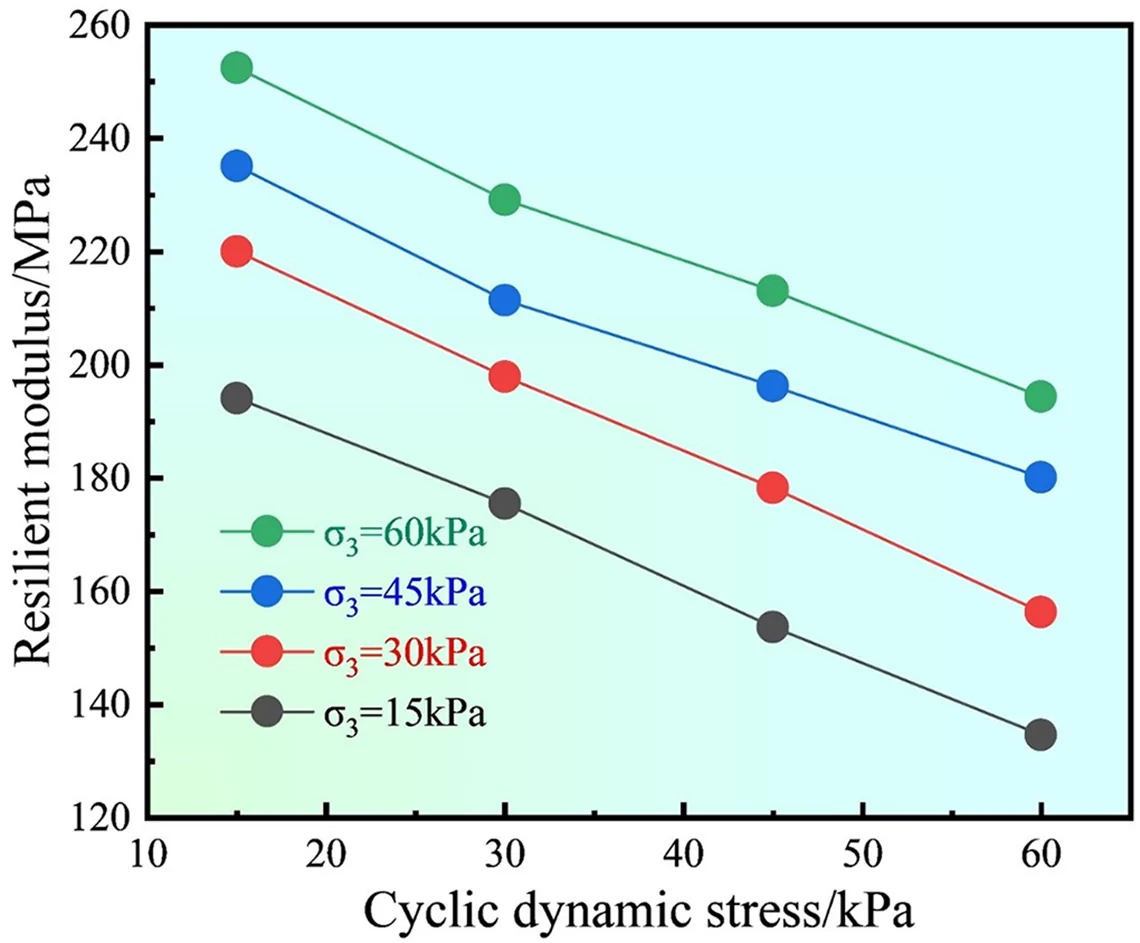
The relationship between M_R_ and $\sigma_{𝐝}$ with different $\sigma_{3}$.

Fig 8 shows the relationship between M_R_ and $\sigma_{\text{d}}$ for C = 7% and N = 3. Similar patterns are observed for other values of N, due to space constraints, these are not shown here. The M_R_ of a specimen is influenced not only by the material itself but is also closely related to the stress conditions under which the test is conducted. As can be seen from the figure, when all other conditions are held constant, the M_R_ decreases continuously as $\sigma_{\text{d}}$ increases, and the rate of decrease is significant. Taking N = 3 and $\sigma_{\text{3}}\;$=30 kPa as an example, the values of $\sigma_{\text{d}}$ at 30 kPa, 55 kPa and 75 kPa are 220.04 MPa, 197.87 MPa and 178.24 MPa respectively, representing decreases of 11.2% and 11.0% respectively, with the rates of decline being relatively similar. This is because cyclic dynamic stress causes changes in the intergranular structure within the specimen, leading to fatigue damage. As a result, the stress in certain areas exceeds the material’s strength limit, causing microcracks to form and propagate within the material. This reduces the specimen’s effective bearing area, thereby diminishing its strength.

### 3.3. Effects of the Dry-Wet Cycle on resilient modulus

**Fig 9:**
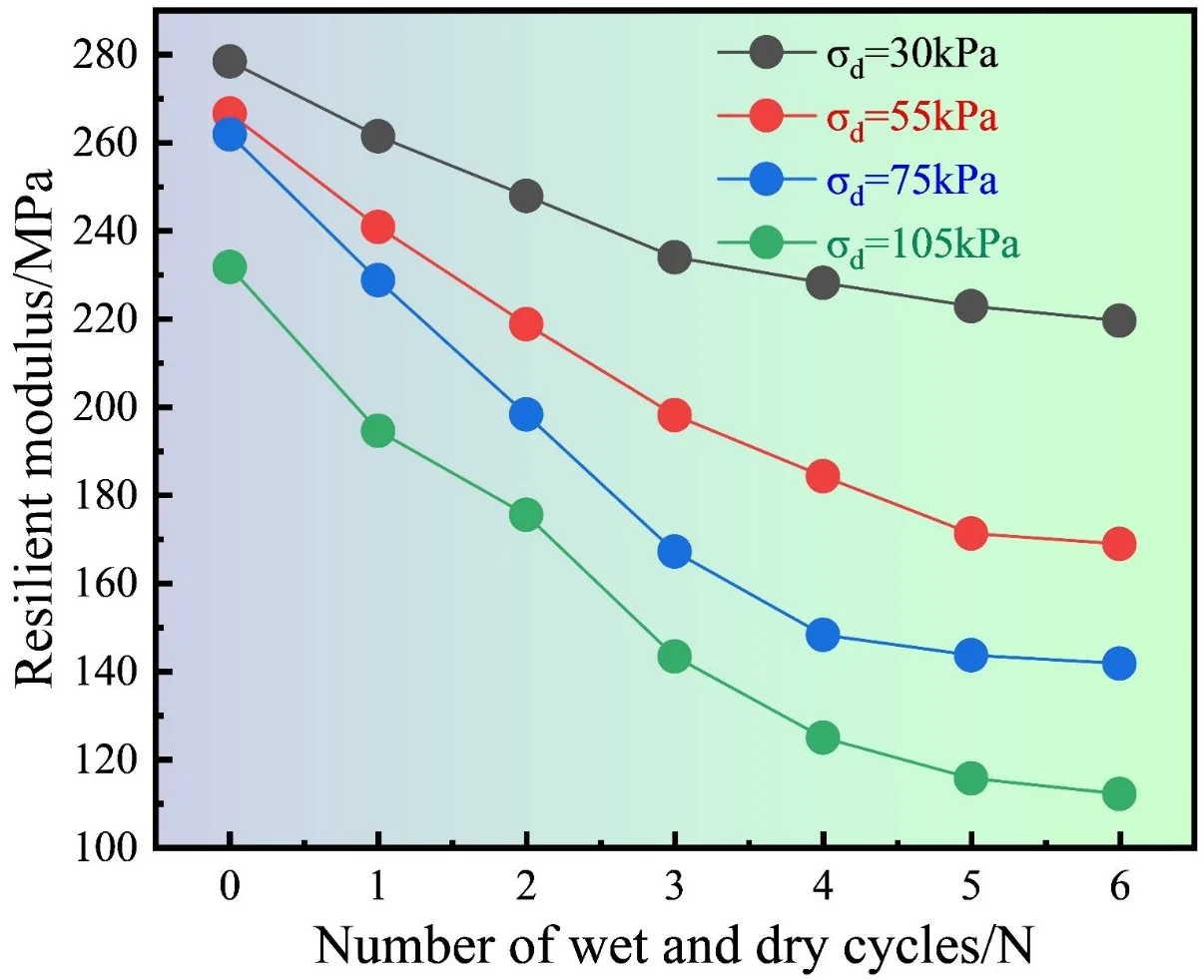
The relationship between M_R_ and N with different $\sigma_{𝐝}$.

The relationship between M_R_ and N at C = 9% and $\sigma_{\text{3}}\;$=15 kPa is shown in Fig 9. Similar patterns were observed for other C; therefore, these are not shown here. The data indicate that prior to the wet-dry cycling, the internal structure of the specimens with the optimal soil conditions remained undisturbed. This happens because when cement-stabilized PG is mixed with water, the cement clinker minerals quickly hydrate to form calcium silicate hydrate (C-S-H gel) and create an alkaline environment; the PG dissolves and releases sulfate ions, which combine with calcium hydroxide and calcium aluminate to form ettringite (AFt). The C-S-H gel and AFt intertwine, not only filling the gaps between particles but also hardening the slurry and forming a three-dimensional network structure, which greatly boosts the strength of the mixture [47].Taking $\sigma_{\text{d}}$ = 55 kPa and N = 0 as an example, the M_R_ is 266.59 MPa; when N = 1, the M_R_ is 240.87 MPa, with a rate of decrease of approximately 9.65%; when N = 4, the M_R_ is 198.12 MPa, and the average rate of decrease over the four trials is 8.81%. When N = 6, M_R_ is 168.89 MPa, and the average rate of decrease for the last two cycles is 4.26%. It can be seen that after one wet-dry cycle, the curve flattens out, the hardening effect gradually weakens, and the soil is damaged under the wet-dry cycles. After 2–4 wet-dry cycles, the damage continues to increase, and internal cracks in the soil develop further, leading to a reduction of M_R_. The decline in M_R_ is mainly concentrated in the first four wet-dry cycles, after which it gradually stabilizes. An analysis of the experimental process suggests that during drying in the oven, temperature differences between the specimen’s surface and interior caused the moisture content to increase gradually from the outside inwards. This led to the generation of residual stresses, inducing tensile stress within the specimen. When the residual stress reached a critical value, cracks formed in the specimen, and subsequent wet-dry cycles exacerbated this phenomenon. However, as the N continues to increase, the development of cracks within the soil stabilizes and the soil reaches a state of equilibrium. Consequently, after four wet-dry cycles, the M_R_ stabilizes.

### 3.4. Effect of Cement dosage on resilient modulus

**Fig 10:**
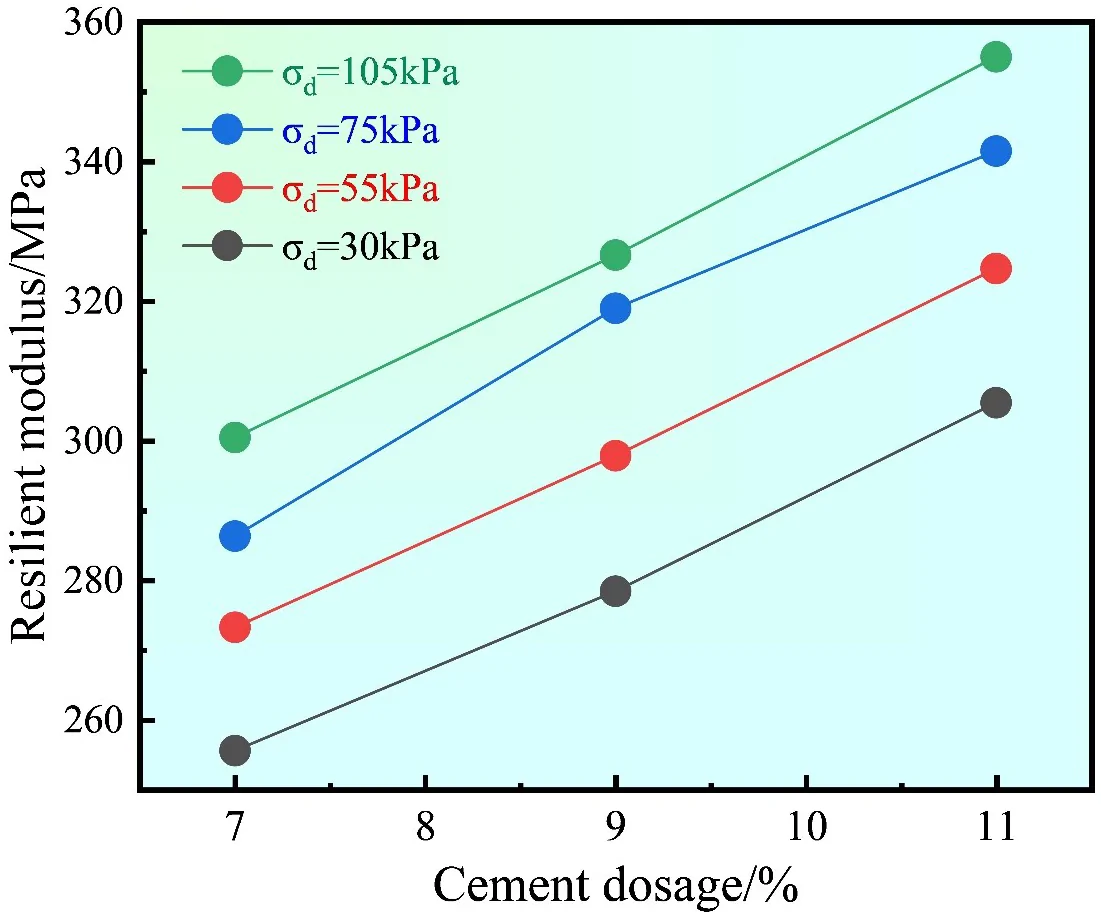
The relationship between d M_R_ and C with different $\sigma_{𝐝}$.


Fig 10 shows the relationship curve of M_R_ and C when $\sigma_{\text{3}}$ = 30 kPa and N = 0. Experimental data reveal a positive correlation between the M_R_ of stabilized materials and C, clearly demonstrating that increased cement dosage effectively enhances the M_R_ of PG. This happens because when cement-stabilized PG is mixed with water, the cement clinker minerals quickly hydrate to form calcium silicate hydrate (C-S-H gel) and create an alkaline environment; the PG dissolves and releases sulfate ions, which combine with calcium hydroxide and calcium aluminate to form ettringite (AFt). The C-S-H gel and AFt intertwine, not only filling the gaps between particles but also hardening the slurry and forming a three-dimensional network structure, which greatly boosts the strength of the mixture [47], as shown in equations (3)-(4) below:

$$\begin{array}{c} \text{3CaO}\cdot \text{Si}O_{\text{2}}+{n\text{H}}_{\text{2}}\text{O}\to x\text{CaO}\cdot \text{Si}O_{\text{2}}\cdot (\text{n}-x-\text{3}){\text{H}}_{\text{2}}\text{O}+(\text{3}-x)\text{3Ca}{\left(\text{OH}\right)}_{\text{2}} \end{array}$$ (3)

$$\begin{array}{c} \text{3CaO}\cdot {\text{Al}}_{\text{2}}{\text{O}}_{\text{3}}+\text{3}\left({\text{CaSO}}_{\text{4}}\cdot \text{2}{\text{H}}_{\text{2}}\text{O}\right)+\text{2}{\text{Ca}(\text{OH})}_{\text{2}}+\text{24 }{\text{H}}_{\text{2}}\text{O}\to \text{3}\text{ CaO}\cdot {\text{Al}}_{\text{2}}{\text{O}}_{\text{3}}\cdot \text{3}{\text{CaSO}}_{\text{4}}\cdot \text{32}{\text{H}}_{\text{2}}\text{O} \end{array}$$ (4)

## 4. Prediction model of resilient modulus

### 4.1. Comparison of resilient modulus and Static resilient Modulus under Dry-Wet Cycling

**Fig 11:**
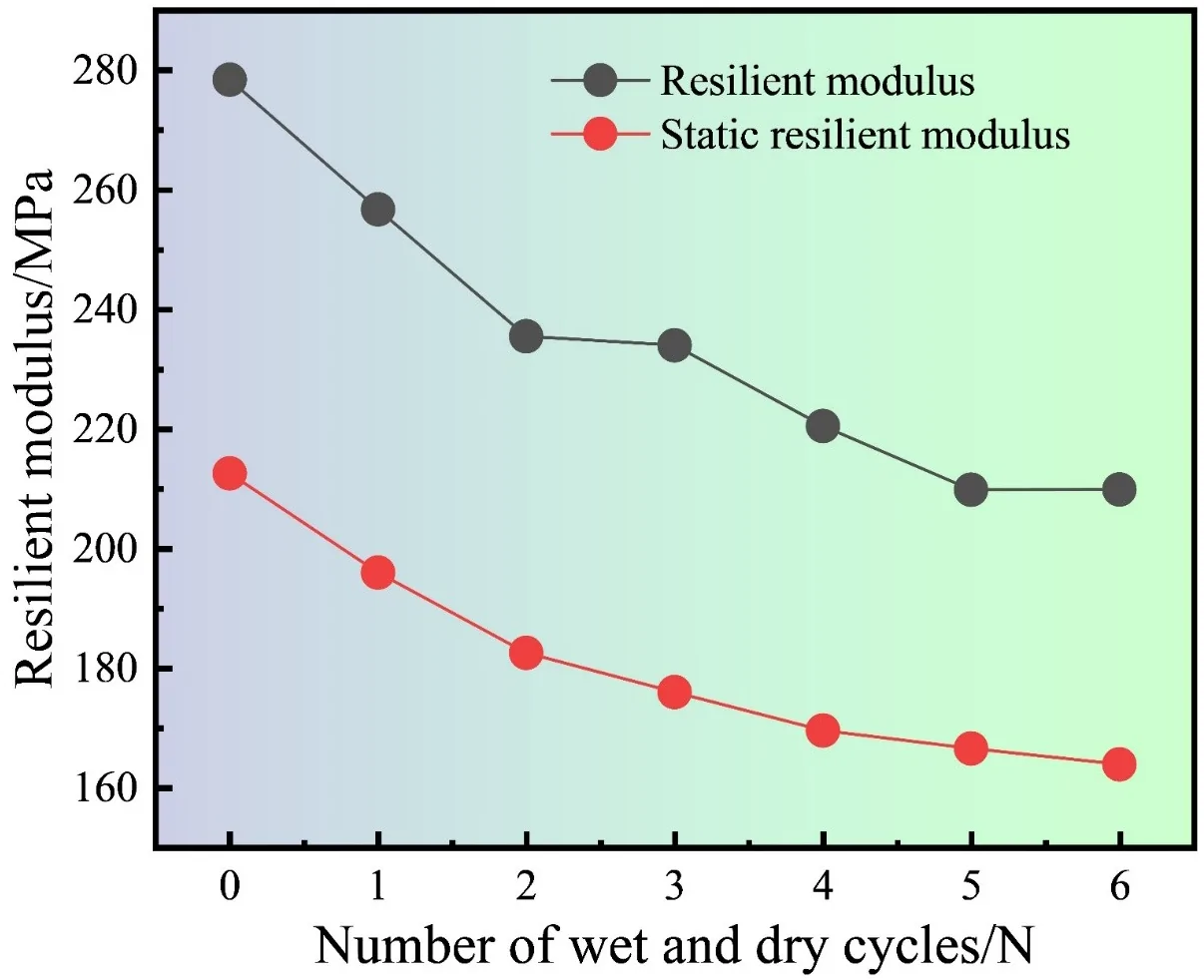
The relationship between M_R_ and static resilient modulus with different N in C = 9%.

Since the measurement of M_R_ has high requirements for test conditions, while the static resilient modulus test is relatively simple, if the proportional relationship between them can be found, M_R_ of the material can be indirectly obtained by measuring the static resilient modulus when M_R_ testing is unavailable. Therefore, this section compares M_R_ and static resilient modulus under dry-wet cycles to provide a reference for obtaining the M_R_ of C-stabilized PG materials. In the test, M_R_ under the most unfavorable conditions of $\sigma_{\text{d}}$=105 kPa and $\sigma_{\text{3}}$=15 kPa was taken as the representative value of C-stabilized PG materials, and the comparison results are shown in Fig 11.

Fig 11 shows the relationship between M_R_ and static resilient modulus at C = 9%. Similar trends are observed at C = 7% and C = 11%, which are not presented here. It can be seen from Fig 11 that both M_R_ and static resilient modulus of the specimens decrease with repeated dry-wet cycles and gradually tend to be stable, and the M_R_ is always much larger than the static value. The variation amplitude of resilient modulus of the specimens becomes very small after 4–6 dry-wet cycles. Therefore, this paper adopts the average values of the last three dry-wet cycles to convert the ratio between them. The representative values of M_R_ and static resilient modulus of specimens with different cement dosage under dry-wet cycles are listed in Table 9. It can be seen from Table 9 that the ratio of M_R_ to static resilient modulus under dry-wet cycles is approximately between 1.25 ~ 1.30. This ratio can be used to make a preliminary estimate of the M_R_ of C-stabilized PG materials, providing a reference for pavement design; however, as the characteristics of PG and local environmental and climatic conditions vary from place to place, further field data is required to verify whether this approach can be widely applied.

**Table 9:** M_R_ and static resilient modulus.

Number of wet and dry cycles	Cement dosage					
	7%		9%		11%	
	M_R_ /(MPa)	Static Values /(MPa)	M_R_ /(MPa)	Static Values /(MPa)	M_R_ /(MPa)	Static Values /(MPa)
0	255.58	193.62	278.43	212.54	305.44	226.25
1	238.76	176.86	256.72	195.97	279.63	211.84
2	202.93	162.34	235.48	182.54	251.42	199.54
3	194.10	151.64	234.00	175.94	242.08	192.65
4	190.79	145.64	220.49	169.54	235.31	186.75
5	180.29	140.85	209.82	166.52	225.59	180.47
6	174.80	137.64	209.85	163.85	222.82	176.84
The last three averages	181.96	141.377	213.387	166.637	227.907	181.353
proportion	1.287		1.281		1.257	

### 4.2. Establishing the Relationship Between resilient modulus and 7-Day Unconfined Compressive Strength

**Fig 12:**
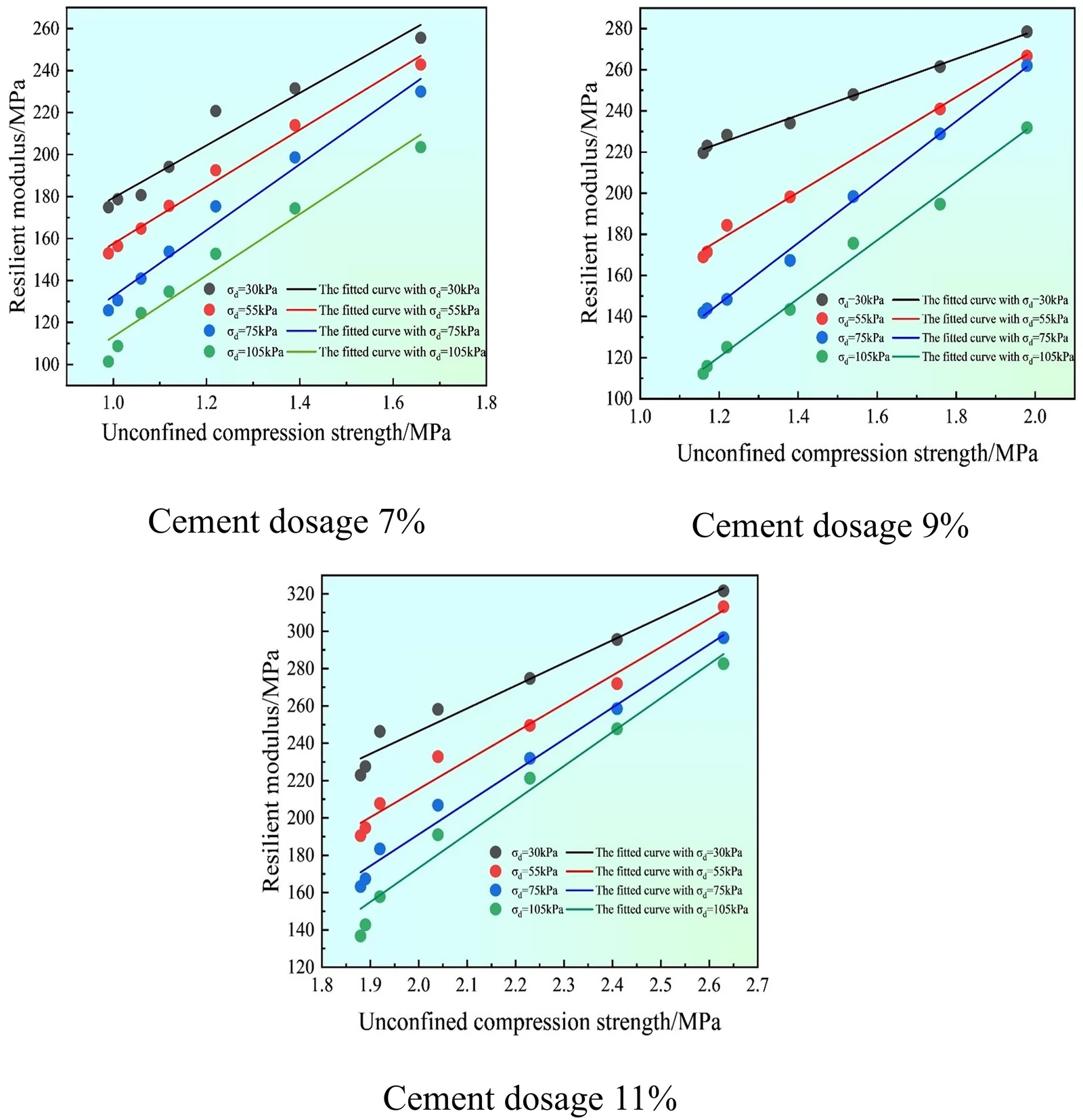
M_R_-$\sigma_{c}$ Fit results.

This paper attempts to establish the relationship between M_R_ and $\sigma_{c}$ for C-stabilized PG materials subjected to wet-dry cycling. The experimental data for M_R_ and $\sigma_{c}$ were fitted using Origin 2018 software; the fitting equation is shown in Equation (5). The results of the fitting curve under the condition of $\sigma_{\text{3}}\;$= 15 kPa are shown in Fig 12. Fig 12 clearly illustrates the degree of deviation between the predicted strength values and the experimental values for specimens with different cement dosage. The fitting results for cement dosage of 7%, 9% and 11% are all satisfactory. Table 10 presents the relevant fitting parameters. As shown in Table 10, the correlation coefficients (R²) are all greater than 0.94, which further demonstrates the high quality of the fitting.

**Table 10:** M_R_-$\sigma_{c}$ Fit parameters.

Cement dosage/(%)	cyclic dynamic stress/(kPa)	a	b	R^2^	RMSE/(MPa)
7%	30	54.23917	124.99127	0.94922	7.021849
	55	21.6653	135.73289	0.98827	3.59139
	75	−24.68569	157.04969	0.9802	5.421966
	105	−32.95036	146.01332	0.97341	6.74496
9%	30	141.69291	68.6059	0.99337	1.780038
	55	38.02405	115.82833	0.99398	2.862542
	75	−50.28235	141.99117	0.99255	2.979097
	105	−31.33448	147.79986	0.99687	2.993069
11%	30	2.77739	121.79095	0.96756	6.498879
	55	−88.63702	152.00592	0.97938	6.428246
	75	−148.202	169.62299	0.98608	5.872735
	105	−190.9979	182.00259	0.98501	9.120252

This model establishes a mathematical relationship between the M_R_ and $\sigma_{c}$ of C-stabilized PG materials under wet-dry cycling. As M_R_ testing imposes high demands on experimental equipment and environmental conditions, this model can indirectly estimate the M_R_ of C-stabilized PG by testing $\sigma_{c}$ under wet-dry cycling, thereby providing a reference for M_R_ prediction and holding practical significance. However, given the variability of on-site conditions, the complexity of engineering scenarios, and the limited sample size of the tests, the application of this model in engineering practice must be supplemented by in-situ testing, long-term monitoring data, and adaptive parameter calibration to further enhance its reliability and engineering applicability under real-world, complex conditions.


$$\begin{array}{c} {\text{M}}_{\text{R}}=a+b\sigma_{c} \end{array}$$ (5)

In the equation: a and b are fitting parameters; $\sigma_{c}$ is the 7-day unconfined compressive strength.

### 4.3. Significance analysis

The analyses presented in Sections 3.1 to 3.4 indicate that $\sigma_{\text{d}}$, $\sigma_{\text{3}}$, C and N have varying degrees of influence on the M_R_ of PG. However, the relative importance of each factor remains unclear. Therefore, it is essential to conduct a sensitivity analysis of these influencing factors before developing an M_R_ prediction model. This analysis will provide valuable insights into the selection of appropriate model parameters, thereby ensuring the accuracy and reliability of the predictive model. A three-factor analysis of variance was conducted using SPSS to evaluate the effects of $\sigma_{\text{d}}$, C and N on the PG mixture, and to analyse the extent to which the three factors$\;\sigma_{\text{d}}$,C and N influence M_R_ when $\sigma_{\text{3}}$=15 kPa. The factor levels and the results of the sensitivity analysis are shown in Tables 11 and 12.

**Table 11:** Test factors and levels.

Levels	$\sigma_{𝐝}$/(kPa)	C/(%)	N/(times)
1	30	7	0
2	55	9	3
3	75	11	6

**Table 12:** Orthogonal analysis results.

Factor	Degree of freedom	Mean square	F	P(significance)
$\sigma_{\text{d}}$	2	2114.053	13.899	0.067
C	2	4211.817	27.690	0.035
N	2	2286.077	15.030	0.062
R^2^ = 0.989				

In Table 12, the smaller the p-value and the larger the F-value, the more significant the influence of that factor on the test results. It can therefore be concluded that there are differences in the extent to which each factor influences the material’s M_R_: The influence intensity of the three factors on the response indicator follows the order of C > N>$\sigma_{\text{d}}$. Factor C exerts a significant effect (P < 0.05), whereas factors N and $\sigma_{\text{d}}$ exhibit marginally significant effects.

### 4.4. Classical prediction mode

In order to quantitatively assess the strength characteristics of subgrade materials under long-term loading, researchers both domestically and internationally have developed a series of M_R_ prediction models, as shown in Table 13. Model 1 considers only $\sigma_{\text{c}}$, Model 2 considers only $\sigma_{\text{d}}$, and Model 3 considers only the confining effect of the volumetric stress θ. Model 4 comprehensively considers both the confining effect of the volumetric stress θ and the influence of the octahedral shear stress $\tau_{\text{oct}}$, providing a more comprehensive description of soil behaviour. The experimental results of this study were fitted using the parameters of typical M_R_ prediction models. However, the overall fitting accuracy was relatively low. This discrepancy arises because classical models contain fewer parameters, making them relatively simple and insufficient to capture the effects of multiple factors.

**Table 13:** Resilient modulus models.

Serial No.	Resilient modulus model	Description
1	${\text{M}}_{\text{R}}={\text{k}}_{\text{1}}{\sigma_{\text{3}}}^{{\text{k}}_{\text{2}}}$	Confining stress Mode [48]
2	${\text{M}}_{\text{R}}={\text{k}}_{\text{1}}{\sigma_{d}}^{{\text{k}}_{\text{2}}}$	Dynamic stress Model [49]
3	${\text{M}}_{\text{R}}={\text{k}}_{\text{1}}\theta^{{\text{k}}_{\text{2}}}$	Bulk stress/k-θ Model [50]
4	${\text{M}}_{\text{R}}={\text{k}}_{\text{1}}{\text{p}}_{\text{a}}{\left(\frac{\theta }{{\text{p}}_{\text{a}}}\right)}^{{\text{k}}_{\text{2}}}{\left(\frac{\tau_{\text{oct}}}{{\text{p}}_{\text{a}}}+\text{1}\right)}^{{\text{k}}_{\text{3}}}$	NCHRP 1–28A Mode [51]

In the given formula. M_R_ denotes the resilient modulus (MPa); $\sigma_{\text{3}}$ represents confining stress (kPa); $\sigma_{d}$ refers to cyclic dynamic stress (kPa); θ is the volumetric stress (kPa); τ_oct_ indicates octahedral shear stress (kPa); p_a_ is typically set to 100 kPa for standard atmospheric pressure; k1, k2, and k3 are fitting parameters that need to be determined.

### 4.5. Estimation model based on wet and dry cycle and cement dosage

Sub-base materials are inevitably subject to wet-dry cycles, which consequently affect their M_R_ values; The traditional prediction models mentioned above do not take this factor into account. Combining the stress conditions and typical stress levels of Chinese subgrades, this section builds upon the NCHRP 1-28A model and, based on experimental data, further incorporates C and N to propose an M_R_ prediction model based on the effects of wet-dry cycling. This model comprehensively considers four factors—C, $\sigma_{\text{d}}$, $\sigma_{\text{3}}$and N—to provide a reference for estimating the M_R_ values of test specimens. By introducing C in exponential form (1 + C)^k^ and N in logarithmic form [ln(e + N)]^k^ into the NCHRP 1-28A model, an improved model is obtained. The use of an exponential function reflects the non-linear enhancing effect of C on the mixture’s bonding properties and overall strength, consistent with the macroscopic mechanical evolution patterns of cement-stabilized materials; The use of the form [ln(e + N)]^k^ reasonably describes the non-linear characteristic whereby material performance gradually decreases and stabilizes under wet-dry cycling. This expression is concise and effectively reflects the actual damage development process of the material. The model is suitable for low-stress conditions; it is not only structurally simple and intuitive but also comprehensively integrates key influencing factors. Furthermore, the parameters of this model have clear physical significance and can be readily obtained through experimental testing. Specifically, as follows:


$$\begin{array}{c} M_{R}=k_{\text{1}}P_{a}{\left(\frac{\theta }{P_{a}}\right)}^{k_{\text{2}}}{\left(\frac{\tau_{oct}}{P_{a}}+\text{1}\right)}^{k_{\text{3}}}(\text{1}+\text{c}{)}^{{\text{k}}_{\text{4}}}{\left[\text{ln}(\text{e}+\text{N})\right]}^{{\text{k}}_{\text{5}}} \end{array}$$ (6)

$$\begin{array}{c} \theta =\sigma_{\text{1}}+\sigma_{\text{2}}+\sigma_{\text{3}} \end{array}$$ (7)

$$\begin{array}{c} \tau_{oct}=\frac{\text{1}}{\text{3}}\sqrt{{\left(\sigma_{\text{1}}-\sigma_{\text{2}}\right)}^{\text{2}}+{\left(\sigma_{\text{1}}-\sigma_{\text{3}}\right)}^{\text{2}}+{\left(\sigma_{\text{2}}-\sigma_{\text{3}}\right)}^{\text{2}}} \end{array}$$ (8)

$$\begin{array}{c} \tau_{oct}=\frac{\sqrt{\text{2}}}{\text{3}}\sigma_{\text{d}}+\text{0}.\text{2}\sigma_{\text{3}} \end{array}$$ (9)

$$\begin{array}{c} \theta =\sigma_{\text{d}}+\text{3}.\text{2}\sigma_{\text{3}} \end{array}$$ (10)

In the given formula, C denotes cement dosage, N represents the number of wet-dry cycles; k_4_, and k_5_ are fitting parameters to be determined. Parameter k_4_ reflects the exponential enhancement effect of cement dosage on M_R_, and k_5_ reflects the extent of strength reduction in the mixture due to wet-dry cycles. $\sigma_{\text{1}}$, $\sigma_{\text{2}}$, and $\sigma_{\text{3}}$ are principal stresses, where $\sigma_{\text{2}}=\sigma_{\text{3}}$, and $\sigma_{\text{1}}=\sigma_{\text{d}}+\text{1}.\text{2}\sigma_{\text{3}}$, yielding Equation (10).

**Fig 13:**
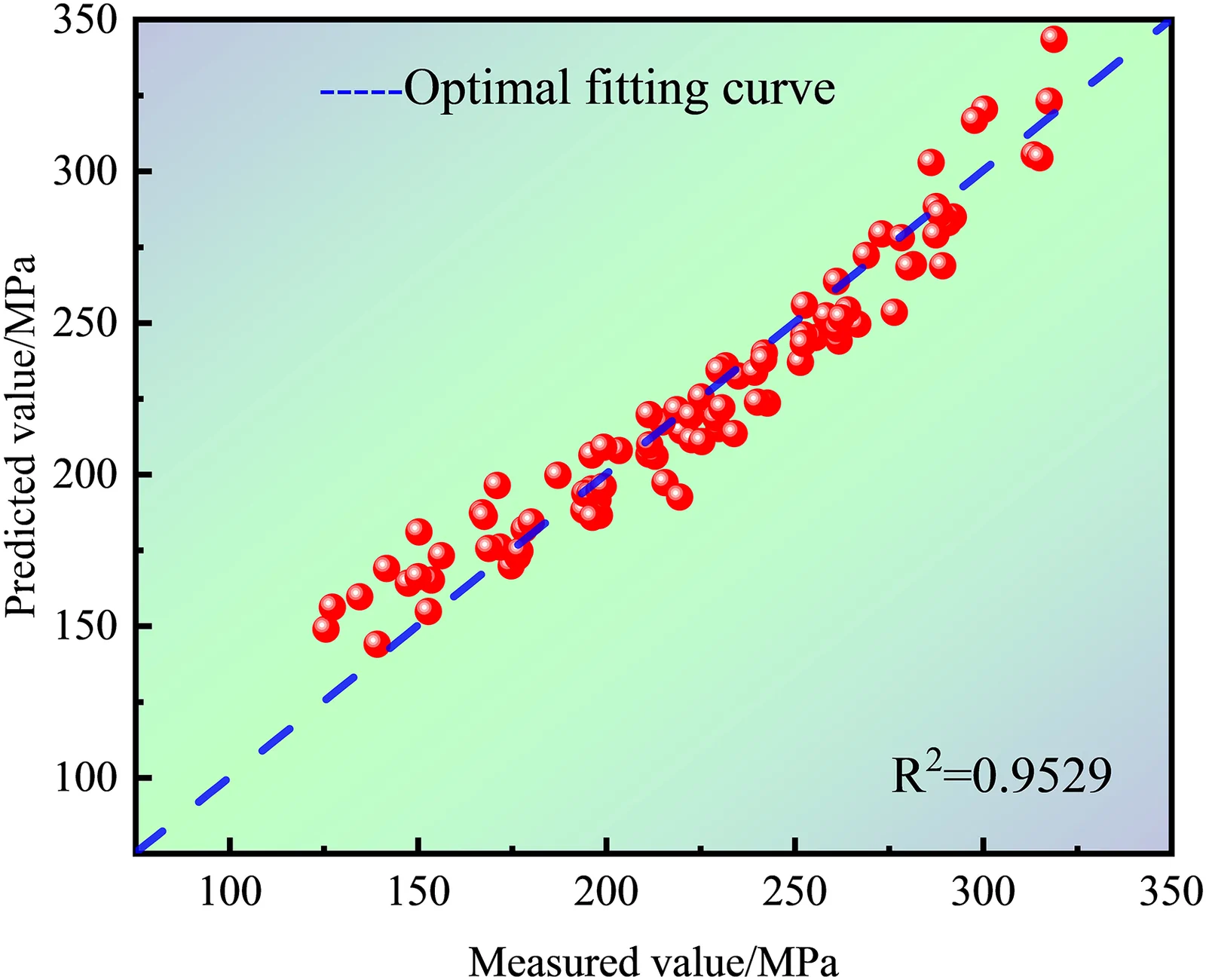
Comparison of measured and predicted values.

By substituting the experimental data for C-stabilized PG materials at N = 0, 3 and 6 into Equation (6), and using a MATLAB programming loop to iterate, the values of k_1_ to k_5_ were obtained, where k_2_ and k_4_ are greater than 0, and k_3_ and k_5_ are less than 0; this is consistent with the conclusions in Sections 3.1 to 3.4. Based on this model, it can be seen that M_R_ increases with an increase in θ ($\sigma_{\text{3}}$) and decreases with an increase in τ_oct_ $\left(\sigma_{\text{d}}\right)$. This is because the weak bonding bonds within the specimen are continuously breaking down, leading to the gradual initiation, propagation and connection of microcracks within the specimen; under repeated loading, the aforementioned damage accumulates continuously, ultimately resulting in a deterioration of the material’s overall stiffness, which manifests macroscopically as a continuous decrease in M_R_. It is noted that the value of k_4_ = 6.8024 is abnormally high, indicating that the test soil is highly sensitive to C. This elevated value is attributed to the combined effect of the selected model form and the material’s inherent non-linearity. Fig 13 was plotted based on the prediction results and experimental data. The MAE_1_ of the prediction results is 13.3, the RMSE_1_ is 17.50, and RD_1_ = 5.6%; the prediction results are satisfactory. To further verify the reliability of the improved model, the MAE_1_, RMSE_1_, and RD_1_ of the M_R_ prediction results under the most unfavorable conditions (C = 7%,$\sigma_{\text{3}}$=15 kPa,$\sigma_{\text{d}}$=105 kPa) for N = 1, 2, 4, and 5 are presented. Table 14 provides the fitted parameter values, the model’s fitting accuracy, and the MAE_2_, RMSE_2_ and RD_2_ of the validation results, where the units for MAE and RMSE are MPa.

**Table 14:** Values of parameters of the proposed modified universal model.

k1	k2	k3	k4	k5	R^2^	MAE_1_	RMSE_1_	RD_1_	MAE_2_	RMSE_2_	RD_2_
0.9129	0.3318	−0.3277	6.8024	−0.4758	0.9529	13.30	17.50	5.60%	12.88	14.20	9.3%

### 4.6. Microscopic mechanism analysis of the mixture under wet-dry cycling

**Fig 14:**
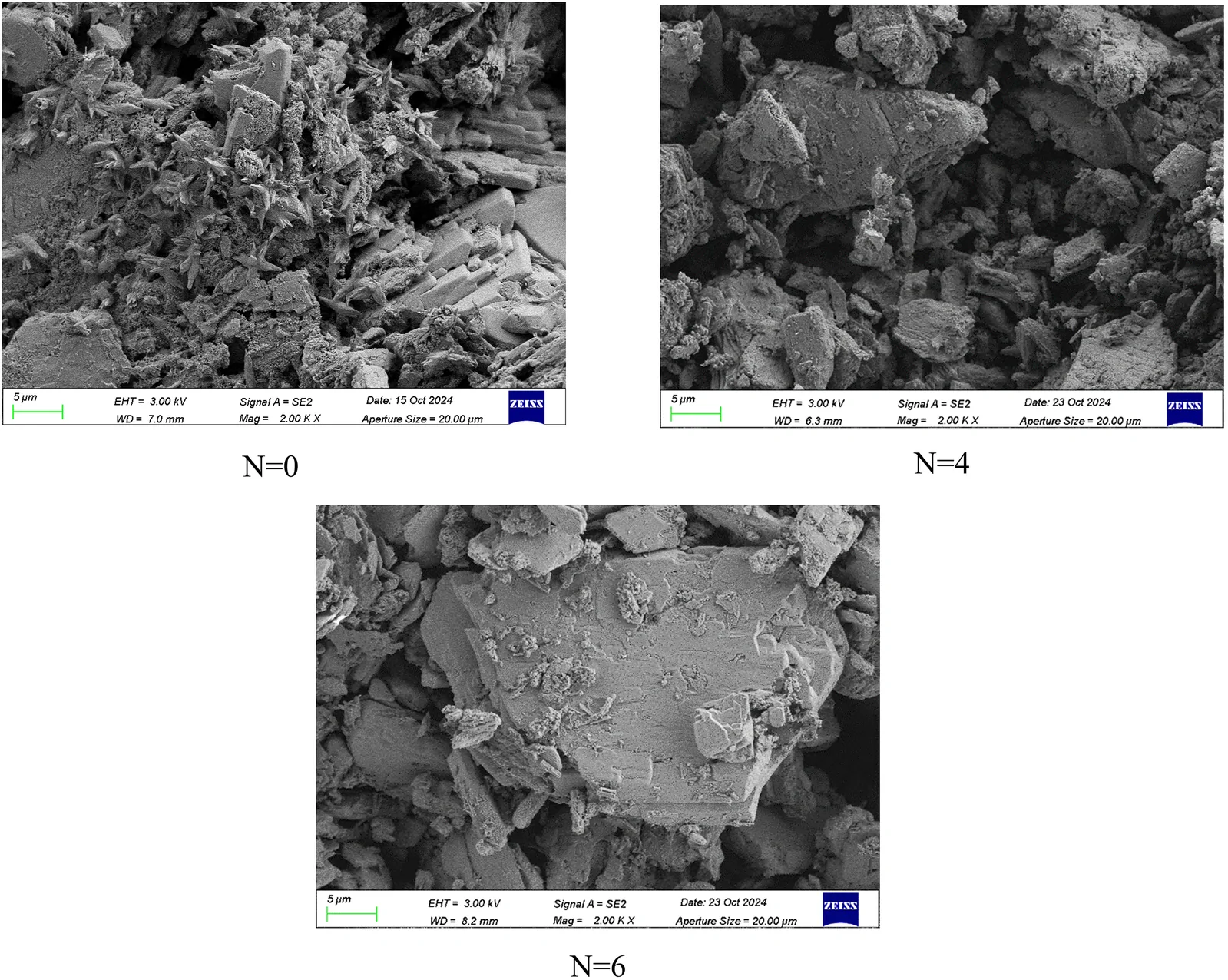
SEM pattern under different N.

**Fig 15:**
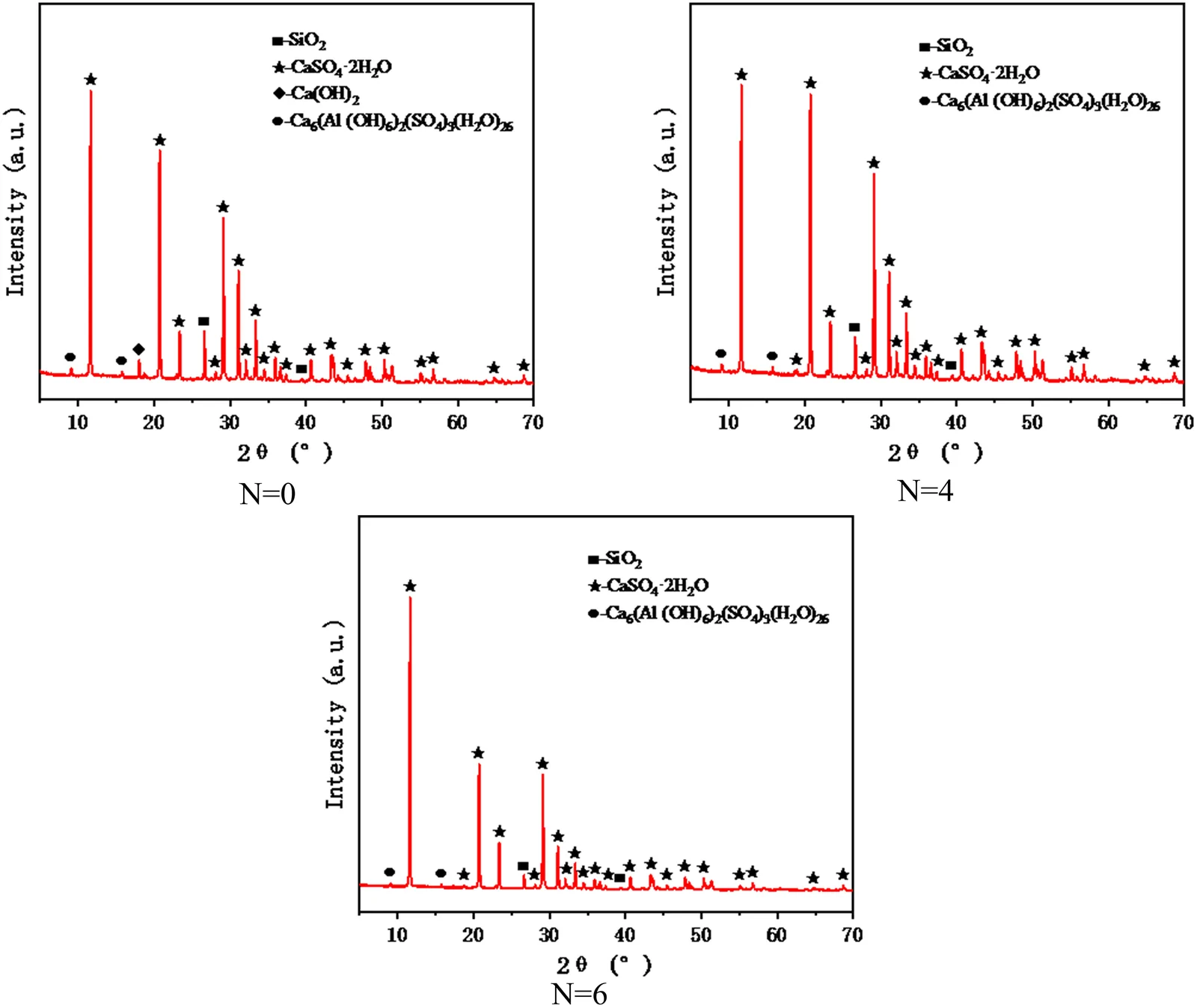
XRD pattern under different N.

To elucidate the mechanisms by which wet-dry cycling affects the properties of the mixture from a microscopic perspective, Figs 14 and 15 present the SEM and XRD micrographs of the C = 9% specimen after 0, 4 and 6 wet-dry cycles, respectively. Observing Fig 14, When N = 0, it can be seen that there is more columnar gel substance between the particles. compared with the specimen that had not undergone wet-dry cycling, after 4 cycles, the content of columnar cementitious material between the lumpy particles was lower, and the inter-particle bonding strength was weaker; when the number of cycles was increased to 6, the pores in the specimens tended to enlarge, and the content of columnar cementitious material between the particles was lower than that observed after 4 cycles. These characteristics of microstructural evolution are consistent with the trend in specimen strength, which initially decreases rapidly and then gradually stabilizes as N increases.

Fig 15 shows that $\text{Ca}{\left(\text{OH}\right)}_{\text{2}}$ disappears in the specimen after 4 ~ 6 dry and wet cycles, the AFt content decreases, and the ${\text{CaSO}}_{\text{4}}\cdot \text{2}{\text{H}}_{\text{2}}\text{O}$ content increases, because part of the AFt decomposes into calcium oxide (CaO), sulfur trioxide (SO_3_) and other substances during the drying process, and SO_3_ dissolved in water to form sulfuric acid (H_2_ SO_4_) during the humidification process, and PG also contains a large amount of ${\text{SO}}_{\text{4}}^{\text{2}-}$, which makes the system acidic. It accelerates the degradation of AFt, which is one of the main reasons for the decrease in AFt in specimens after wet and dry cycling [52,53]. ${\text{CaSO}}_{\text{4}}\cdot \text{2}{\text{H}}_{\text{2}}\text{O}$ content increases and $\text{Ca}{\left(\text{OH}\right)}_{\text{2}}$ disappears, and it is speculated that the possible reason is that the SO_3_ obtained by AFt decomposition reacts with $\text{Ca}{\left(\text{OH}\right)}_{\text{2}}$ after dissolving in water to form ${\text{CaSO}}_{\text{4}}\cdot \text{2}{\text{H}}_{\text{2}}\text{O}$, and the reaction process is as follows:


$$\begin{array}{c} \text{S}{\text{O}}_{\text{3}}+{\text{H}}_{\text{2}}\text{O}+\text{Ca}{\left(\text{OH}\right)}_{\text{2}}={\text{CaSO}}_{\text{4}}\cdot \text{2}{\text{H}}_{\text{2}}\text{O} \end{array}$$ (11)

The main mechanisms of strength attenuation of cement PG stabilized materials under the above dry and wet cycles are as follows: (1) The dry and wet cycle increases the porosity inside the specimen, reduces the cementitious matter between the plates, weakens the connection effect, and reduces the integrity of the specimen. (2) The dry and wet cycle partially decomposes AFt, and the reinforcement effect of AFt is weakened.

## 5. Conclusions

This study investigates the variation patterns of the M_R_ of cement-stabilized PG materials under different conditions through dynamic triaxial tests, analyzes the mechanism behind M_R_ reduction, and focuses on the mechanical properties of PG mixtures in regions with seasonal rainfall. Through a series of dynamic triaxial experiments and microscopic analyses, the microstructural characteristics of specimens were examined, and an M_R_ prediction model was established for different cement dosages under wet-dry cycling conditions. The main conclusions are as follows:

(1) The M_R_ of stabilized materials increases with higher confining stress and cement dosage, while gradually decreasing with increasing cyclic dynamic stress. Under wet-dry cycles, the M_R_ decline of PG mixtures undergoes three stages: rapid decay, moderate decay, and stable stage. The most pronounced M_R_ decline occurs during the first cycle. As the number of wet-dry cycles increases, M_R_ continues to decrease but at a slower rate, stabilizing after approximately four cycles. And through a microscopic explanation of its causes, the wet-dry cycles increase the porosity inside the specimen, reduce the cementitious material between the plates, weaken the bonding effect, and decrease the overall integrity of the specimen; the wet-dry cycles cause partial decomposition of AFt, weakening the reinforcing effect of AFt.
(2) Under dry-wet cycling, the relationship between M_R_ of stabilized materials and their 7-day unconfined compressive strength can be described by M_R_ = a + b$\sigma_{\text{c}}$. The correlation coefficients for fitting exceed 0.94, indicating good fitting quality. This validates the reliability of estimating the M_R_ of stabilized materials using 7-day unconfined compressive strength, providing a reference for studying the strength characteristics of cement-stabilized PG materials.
(3) Comparing dynamic and static resilient modulus reveals that the M_R_ of PG specimens under wet-dry cycling is 1.25–1.30 times the static resilient modulus. This ratio can be used to estimate the M_R_ of cement-stabilized PG materials. Of course, whether it can be rolled out for wider use will require further verification through field data.
(4) Multi-factor sensitivity analysis indicates that C and N influence the mixture’s M_R_. Therefore, a M_R_ prediction model incorporating wet-dry cycling effects and cement dosage is proposed: ${\text{M}}_{\text{R}}={\text{k}}_{\text{1}}{\text{p}}_{\text{a}}(\theta /\text{pa}{)}^{{\text{k}}_{\text{2}}}{\left(\tau_{\text{oct}}/p_{a}+\text{1}\right)}^{{\text{k}}_{\text{3}}}{\left(\text{1}+\text{C}\right)}^{{\text{k}}_{\text{4}}}{\left[\text{ln}(\text{e}+\text{N})\right]}^{{\text{k}}_{\text{5}}}$. The model’s reliability was validated by comparing predicted values with experimental data. This model is no longer limited to simple statements at the phenomenological level, but rather, through function fitting and pattern analysis, it extracts the nonlinear evolution mechanism of modulus changes with influencing factors, thereby deepening the understanding of the intrinsic mechanical response of cement-stabilized PG materials under complex working conditions
(5) This experiment fully utilizes the readily available $\sigma_{c}$ and static resilient modulus, and establishes predictive models of C and N with respect to M_R_. By comparing measured values with model predictions, the reliability of the models is verified, providing a new approach for predicting M_R_. Additionally, microscopic experiments are used to explain the reasons for M_R_ reduction under wet-dry cycles. Although actual road engineering conditions are relatively complex, with multi-factor coupling phenomena, this experiment is a reasonable simplification of field complexity under controlled conditions. The conclusions obtained have certain engineering value.
(6) This study creatively proposes a M_R_ prediction model that considers the effects of wet-dry cycles and cement dosage, and validates the reliability of the model by comparing predicted values with experimental results. The predicted M_R_ provides a reliable basis for pavement design and holds significant engineering guidance value. Because of the variability of onsite conditions and the complexity of engineering situations, and given that our test sample size is limited, this model, when applied to actual projects, still needs to be combined with on-site testing, long-term monitoring data, and adaptive parameter calibration to further improve its reliability and suitability under real, complex conditions. We hope more researchers will carry out in-depth analyses on the environmental safety of PG, testing the leachate of cement-stabilized PG samples under wet-dry cycles for radioactivity, heavy metal content, and pH value, to ensure the environmental safety of PG when exposed onsite.

## Supporting information

S1 File Minimum Dataset. (ZIP)
