# Supplementary material for: Resilient modulus characteristics and model prediction of cement phosphogypsum under dry-wet cycling conditions
Source: PLoS One. 2026 Sep 8;21(9):e0341539. doi: 10.1371/journal.pone.0341539 (PMC13552755; doi:10.1371/journal.pone.0341539)
Supplement: Supplementary file 1 [file pone.0341539.s001.zip › Supporting Information/MATLAB parameter fitting code/MATLAB parameter fitting code/Fitting parameters/Find the fitting parameters.docx]

$$M_{R}=k_{1}p_{a}\left( \frac{\theta}{p_{a}} \right)^{k_{2}}\left( \frac{\tau_{oct}}{p_{a}}+1 \right)^{k_{3}}{(1+c)}^{k_{4}}\left[ ln(e+N) \right]^{k_{5}}$$

In the given formula, M_R_ denotes the M_R_ (kPa);；

θ is the bulk stress (kPa), $\theta=\sigma_{1}+\sigma_{2}+\sigma_{3}$；

τ_oct_ indicates octahedral shear stress (kPa)；

$\tau_{oct}=\frac{1}{3}\sqrt{{{(\sigma}_{1}-\sigma_{2})}^{2}+{{(\sigma}_{1}-\sigma_{3})}^{2}{{+(\sigma}_{2}-\sigma_{3})}^{2}}$；

$\sigma_{1}$、$\sigma_{2}$、$\sigma_{3}$ are principal stresses，$\sigma_{2}=\sigma_{3}$，$\sigma_{1}=\sigma_{d}+1.2\sigma_{3}$；

$N$ represents the number of wet-dry cycles；

k₁, k₂, k₃, k₄, and k₅ are fitting parameters to be determined；

p_a_ is typically set to 100 kPa for standard atmospheric pressure;
