# Supplementary material for: Resilient modulus characteristics and model prediction of cement phosphogypsum under dry-wet cycling conditions
Source: PLoS One. 2026 Sep 8;21(9):e0341539. doi: 10.1371/journal.pone.0341539 (PMC13552755; doi:10.1371/journal.pone.0341539)
Supplement: Supplementary file 1 [file pone.0341539.s001.zip › Supporting Information/Minimun data set.docx]

### Resilient modulus Characteristics and Model Prediction of Cement Phosphogypsum under Dry-Wet Cycling Conditions

Wengang Gan^1^, Kaisheng Chen^1*^, Jinxiong Chen^1^,Yu'ang Chen^2^, Shuang Deng^3^

(1.College of Civil Engineering, Guizhou University, Guiyang 550025, China; 2. School of Civil Engineering, Southwest Jiaotong University, Chengdu 611756,, China, 3. School of Architectural Engineering, Kaili University, Kaili, Guizhou 556011, China)

Table 1. Chemical Composition of PG.

| Ingredient | SO_3_ | CaO | SiO_2_ | P_2_O_5_ | Na_2_O | Al_2_O_3_ | Other |
| --- | --- | --- | --- | --- | --- | --- | --- |
| Mass fraction/(%) | 50.44 | 39.26 | 6.05 | 1.47 | 0.49 | 0.55 | 1.74 |

Table 2. Basic physical parameters of PG.

| Wet density  / (g∙cm-3) | Dry density  / (g∙cm-3) | Water content/(%) | Liquid limit ($w_{l}$) /(%) | Plastic limit ($w_{p}$) / (%) | Plasticity index ($I_{p}$) / (%) |
| --- | --- | --- | --- | --- | --- |
| 1.23 | 1.07 | 14.5 | 27.5 | 13.7 | 13.8 |

Table 3. PG radioactivity test results.

| Testing items | Technical requirements: | Test results | Single decision | Detection conclusion |
| --- | --- | --- | --- | --- |
| Cra (Bq/kg) | / | 53.89 | / | Complies with the requirements of the national standard GB 6566-2010 “Limits for Radionuclides in Building Materials” |
| CTh (Bq/kg) | / | 42.09 | / |  |
| CK(Bq/kg) | / | 53.89 | / |  |
| Internal irradiation index(Ira) | ≤1.0 | 0.4 | Qualified |  |
| External beam index (Ir) | ≤1.0 | 0.4 | Qualified |  |

Table 4. Test results of hazardous components in PG leachate.

| Testing items | Technical requirements: | Test results | Single decision | Detection conclusion |
| --- | --- | --- | --- | --- |
| Cu(mg/L) | 100 | 0.157 | Qualified | It meets the requirements of GB 5085.3-2007 "Identification of Hazardous Waste Identification Standards for Leaching Toxicity" |
| Zn(mg/L) | 100 | 0.051 | Qualified |  |
| Cd(mg/L) | 1 | - | Qualified |  |
| Pb(mg/L) | 5 | - | Qualified |  |
| Cr(mg/L) | 15 | - | Qualified |  |
| As(mg/L) | 5 | 0.0361 | Qualified |  |
| Hg(mg/L) | 0.1 | 0.0006 | Qualified |  |

Table 5. Basic parameters of cement.

| setting time /(min) | | $f_{cf}$/(MPa) | | $f_{cu}$/(MPa) | |
| --- | --- | --- | --- | --- | --- |
| Initial | Final | 3d | 28d | 3d | 28d |
| 302 | 322 | 5.9 | 6.7 | 24.9 | 43.7 |

Table 6. Chemical Composition of PG of cement.

| Ingredient | CaO | SiO_2_ | Al_2_O_3_ | Fe_2_O_3_ | MgO | 其他 |
| --- | --- | --- | --- | --- | --- | --- |
| Mass fraction/(%) | 63.5 | 22.4 | 4.5 | 3.3 | 2.2 | 4.1 |

**Table 7. Cement phosphogypsum compaction test results.**

| Cement dosage /(%) | Compaction/(%) | Maximum Dry Density /(g∙cm^-3^) | Optimum Moisture Content/(%) |
| --- | --- | --- | --- |
| 7% | 98% | 1.515 | 14.56 |
| 9% |  | 1.486 | 14.01 |
| 11% |  | 1.502 | 14.66 |

Table 8. Loading sequence of fine-grained soil specimens.

| Load the serial number | confining stress $\sigma_{3}$/(Kpa) | Contact stress $\sigma_{c1}$/(Kpa) | cyclic dynamic stress  $\sigma_{d}$/(Kpa) | axial stress  $\sigma_{max}$/(Kpa) | The number of loads acted |
| --- | --- | --- | --- | --- | --- |
| 0 | 30 | 6 | 55 | 61 | 1000 |
| 1 | 60 | 12 | 30 | 42 | 100 |
| 2 | 45 | 9 | 30 | 39 | 100 |
| 3 | 30 | 6 | 30 | 36 | 100 |
| 4 | 15 | 3 | 30 | 33 | 100 |
| 5 | 60 | 12 | 55 | 67 | 100 |
| 6 | 45 | 9 | 55 | 64 | 100 |
| 7 | 30 | 6 | 55 | 61 | 100 |
| 8 | 15 | 3 | 55 | 58 | 100 |
| 9 | 60 | 12 | 75 | 87 | 100 |
| 10 | 45 | 9 | 75 | 84 | 100 |
| 11 | 30 | 6 | 75 | 81 | 100 |
| 12 | 15 | 3 | 75 | 78 | 100 |
| 13 | 60 | 12 | 105 | 117 | 100 |
| 14 | 45 | 9 | 105 | 114 | 100 |
| 15 | 30 | 6 | 105 | 111 | 100 |
| 16 | 15 | 3 | 105 | 108 | 100 |

Table 9. M_R_ and static resilient modulus.

| Cement  dosage  Number  of wet and  dry cycles | 7% | | 9% | | 11% | |
| --- | --- | --- | --- | --- | --- | --- |
|  | M_R_  /(MPa) | Static  Values  /(MPa) | M_R_  /(MPa)） | Static  Values  /(MPa) | M_R_  /(MPa) | Static  Values  /(MPa) |
| 0 | 255.58 | 193.62 | 278.43 | 212.54 | 305.44 | 226.25 |
| 1 | 238.76 | 176.86 | 256.72 | 195.97 | 279.63 | 211.84 |
| 2 | 202.93 | 162.34 | 235.48 | 182.54 | 251.42 | 199.54 |
| 3 | 194.10 | 151.64 | 234.00 | 175.94 | 242.08 | 192.65 |
| 4 | 190.79 | 145.64 | 220.49 | 169.54 | 235.31 | 186.75 |
| 5 | 180.29 | 140.85 | 209.82 | 166.52 | 225.59 | 180.47 |
| 6 | 174.80 | 137.64 | 209.85 | 163.85 | 222.82 | 176.84 |
| The last  three averages | 181.96 | 141.377 | 213.387 | 166.637 | 227.904 | 181.353 |
| proportion | 1.287 | | 1.281 | | 1.257 | |

Table 10. M_R_-σ_c_ Fit parameters.

| Cement  dosage /(%) | cyclic dynamic  stress/(kPa) | a | b | R^2^ | RMSE/(MPa) |
| --- | --- | --- | --- | --- | --- |
| 7% | 30 | 54.23917 | 124.99127 | 0.94922 | 7.021849 |
|  | 55 | 21.6653 | 135.73289 | 0.98827 | 3.59139 |
|  | 75 | -24.68569 | 157.04969 | 0.9802 | 5.421966 |
|  | 105 | -32.95036 | 146.01332 | 0.97341 | 6.74496 |
| 9% | 30 | 141.69291 | 68.6059 | 0.99337 | 1.780038 |
|  | 55 | 38.02405 | 115.82833 | 0.99398 | 2.862542 |
|  | 75 | -50.28235 | 141.99117 | 0.99255 | 2.979097 |
|  | 105 | -31.33448 | 147.79986 | 0.99687 | 2.993069 |
| 11% | 30 | 2.77739 | 121.79095 | 0.96756 | 6.498879 |
|  | 55 | -88.63702 | 152.00592 | 0.97938 | 6.428246 |
|  | 75 | -148.202 | 169.62299 | 0.98608 | 5.872735 |
|  | 105 | -190.9979 | 182.00259 | 0.98501 | 9.120252 |

Table 11. Test factors and levels.

| Levels | $\sigma_{d}$/(kPa) | C/(%) | N/(times) |
| --- | --- | --- | --- |
| 1 | 30 | 7 | 0 |
| 2 | 55 | 9 | 3 |
| 3 | 75 | 11 | 6 |

Table 12. Orthogonal analysis results.

| Factor | Degree of freedom | Mean square | F | Significance |
| --- | --- | --- | --- | --- |
| $\sigma_{d}$ | 2 | 2114.053 | 13.899 | 0.067 |
| C | 2 | 4211.817 | 27.690 | 0.035 |
| N | 2 | 2286.077 | 15.030 | 0.062 |
| R^2^=0.989 |  |  |  |  |

Orthogonal Test Design Results

| $\sigma_{3}$/(kPa) | $\sigma_{d}$/(kPa) | C | N | M_R_/(MPa) |
| --- | --- | --- | --- | --- |
| 15 | 1 | 1 | 1 | 230.41 |
| 15 | 1 | 2 | 2 | 210.34 |
| 15 | 1 | 3 | 3 | 252.16 |
| 15 | 2 | 1 | 2 | 148.45 |
| 15 | 2 | 2 | 3 | 168.16 |
| 15 | 2 | 3 | 1 | 298.06 |
| 15 | 3 | 1 | 3 | 125.65 |
| 15 | 3 | 2 | 2 | 156.14 |
| 15 | 3 | 3 | 1 | 251.86 |

Table 13. Resilient modulus models.

| Serial No. | Resilient modulus model | Description |
| --- | --- | --- |
| 1 | $M_{R}=k_{1}{\sigma_{3}}^{k_{2}}$ | Confining pressure Mode |
| 2 | $M_{R}=k_{1}{\sigma_{d}}^{k_{2}}$ | Dynamic stress Model |
| 3 | $M_{R}=k_{1}\theta^{k_{2}}$ | Bulk stress/k-θ Model |
| 4 | $M_{R}=k_{1}p_{a}\left( \frac{\theta}{p_{a}} \right)^{k_{2}}\left( \frac{\tau_{\mathrm{oct}}}{p_{a}}+1 \right)^{k_{3}}$ | NCHRP 1–28A Mode |

Table 14. Values of parameters of the proposed modified universal model.

| C=7%、$\sigma_{d}=$15Kpa_、_$\sigma_{d}$=105Kpa | | | | | |
| --- | --- | --- | --- | --- | --- |
| N | Experimental value /(Mpa) | Predicted value/(Mpa) | MAE_2_/(Mpa) | RMSE_2_/(Mpa) | RD_2_//(Mpa) |
| 1 | 174.25 | 220.04 | 12.88 | 14.20 | 9.3% |
| 2 | 152.64 | 197.87 | 12.88 | 14.20 | 9.3% |
| 4 | 153.64 | 178.24 | 12.88 | 14.20 | 9.3% |
| 5 | 134.58 | 156.28 | 12.88 | 14.20 | 9.3% |

Fig 1. PG used in the test.

Fig 2. PG particle grading curve.

| Sinking time /min | Soil particle settling distance/(L/cm) | Particle sized/mm | Mass percentage of soil smaller than a certain particle sizeX/% |
| --- | --- | --- | --- |
| 0.5 | 11.52 | 0.0713 | 100 |
| 1 | 13.64 | 0.0548 | 96.95 |
| 5 | 13.97 | 0.0248 | 78.3 |
| 15 | 14.39 | 0.0145 | 64.43 |
| 30 | 14.86 | 0.0104 | 52.56 |
| 60 | 15.51 | 0.0075 | 33.7 |
| 120 | 15.62 | 0.0054 | 15.4 |
| 240 | 16.19 | 0.0039 | 8.48 |

Fig 3. Flowchart of the testing process.

Fig 4. Water content and dry density curve under under C=9%..

| C=9% | | | |
| --- | --- | --- | --- |
| Moisture content/(%) | Dry density/(g∙cm^-3^) | Optimal moisture content/(%) | Maximum dry density/(g∙cm^-3^) |
| 9.92% | 1.12 | 13.01% | 1.486 |
| 11.46% | 1.33 |  |  |
| 14.46% | 1.48 |  |  |
| 15.74% | 1.42 |  |  |
| 17.36% | 1.23 |  |  |

Fig 5. Sample preparation scheme and strength test.

Fig 6. Specimen preparation process.

Fig 7. The relationship between M_R_ and $\sigma_{3}$ with different $\sigma_{d}$.

| N=6、C=7% | | | | |
| --- | --- | --- | --- | --- |
| $\sigma_{d}$/(MPa) | | | | |
| $\sigma_{3}/(MPa)$ | 30 | 55 | 75 | 105 |
| 15 | 174.80 | 152.84 | 125.65 | 101.25 |
| 30 | 195.68 | 176.48 | 147.48 | 127.32 |
| 45 | 211.61 | 198.24 | 171.89 | 150.14 |
| 60 | 230.78 | 215.64 | 196.35 | 177.15 |

Fig 8. The relationship between M_R_ and $\sigma_{d}$ with different $\sigma_{3}$.

| N=3、C=7% | | | | |
| --- | --- | --- | --- | --- |
| $\sigma_{d}$/(MPa) | | | | |
| $\sigma_{d}$/(MPa) | 15 | 30 | 45 | 60 |
| 30 | 194.10 | 220.04 | 235.14 | 252.48 |
| 55 | 175.48 | 197.87 | 211.41 | 229.14 |
| 75 | 153.64 | 178.24 | 196.21 | 213.04 |
| 105 | 134.58 | 156.284 | 180.11 | 194.35 |

Fig 9. The relationship between M_R_ and N with different $\sigma_{d}$.

| C=9%、$\sigma_{3}$=15KPa | | | | |
| --- | --- | --- | --- | --- |
| $\sigma_{d}$/(MPa) | | | | |
| N | 30 | 55 | 75 | 105 |
| 0 | 278.42 | 276.59 | 261.96 | 231.76 |
| 1 | 261.45 | 240.87 | 228.76 | 194.56 |
| 2 | 247.89 | 218.79 | 198.28 | 175.48 |
| 3 | 234.01 | 198.12 | 167.16 | 150.32 |
| 4 | 228.15 | 184.26 | 148.25 | 124.89 |
| 5 | 222.84 | 171.25 | 143.61 | 115.65 |
| 6 | 219.56 | 168.89 | 141.73 | 112.08 |

Fig 10. The relationship between d M_R_ and C with different $\sigma_{d}$.

| $\sigma_{d}$=30Kpa、N=0 | | | | |
| --- | --- | --- | --- | --- |
|  | $\sigma_{3}$/(MPa) | | | |
| C | 15 | 30 | 45 | 60 |
| 7 | 255.58 | 273.26 | 286.32 | 300.47 |
| 9 | 278.43 | 297.85 | 318.96 | 326.61 |
| 11 | 305.44 | 324.66 | 341.52 | 355.02 |

Fig 11. The relationship between the M_R_ and static resilient modulus with different N.

|  | C=9% | |
| --- | --- | --- |
| N | M_R_/(MPa） | Static value/(MPa) |
| 0 | 278.43 | 212.54 |
| 1 | 256.72 | 195.97 |
| 2 | 235.48 | 182.54 |
| 3 | 234.87 | 175.94 |
| 4 | 220.49 | 169.54 |
| 5 | 209.82 | 166.52 |
| 6 | 209.85 | 163.85 |
| Average of the last three times | 218.287 | 166.637 |
| Dynamic/Static | 1.281 | |

|  |  |
| --- | --- |
| Cement admixture 7% | Cement admixture 9% |
|  | |
| Cement admixture 11% | |

Fig 12. M_R_-$\sigma_{c}$ Fit results.

| 7-day unconfined compressive strength | | | | | | | |
| --- | --- | --- | --- | --- | --- | --- | --- |
|  | N | | | | | | |
| C/(%) | 0 | 1 | 2 | 3 | 4 | 5 | 6 |
| 7% | 1.66 | 1.39 | 1.22 | 1.12 | 1.06 | 1.01 | 0.99 |
| 9% | 1.98 | 1.76 | 1.54 | 1.38 | 1.22 | 1.17 | 1.16 |
| 11% | 2.63 | 2.41 | 2.23 | 2.04 | 1.92 | 1.89 | 1.88 |

| C=7%、$\sigma_{3}=$15KPa | | | | |
| --- | --- | --- | --- | --- |
|  | $\sigma_{d}$/(MPa) | | | |
| N | 30 | 55 | 75 | 105 |
| 0 | 255.584 | 242.84 | 229.94 | 203.48 |
| 1 | 231.46 | 213.98 | 198.56 | 174.25 |
| 2 | 220.68 | 192.45 | 175.25 | 152.64 |
| 3 | 194.11 | 175.48 | 153.64 | 134.58 |
| 4 | 180.58 | 164.65 | 140.78 | 124.32 |
| 5 | 178.65 | 156.36 | 130.45 | 108.64 |
| 6 | 174.85 | 152.84 | 125.65 | 101.25 |

| C=9%、$\sigma_{3}=$15KPa | | | | |
| --- | --- | --- | --- | --- |
|  | $\sigma_{d}$/(MPa) | | | |
| N | 30 | 55 | 75 | 105 |
| 0 | 278.43 | 266.59 | 261.92 | 231.76 |
| 1 | 261.45 | 240.87 | 228.76 | 194.56 |
| 2 | 247.89 | 218.79 | 198.28 | 175.48 |
| 3 | 234.05 | 198.12 | 167.16 | 143.33 |
| 4 | 228.15 | 184.26 | 148.25 | 124.89 |
| 5 | 222.84 | 171.25 | 143.61 | 115.65 |
| 6 | 219.56 | 168.88 | 141.73 | 112.08 |

| C=11%、$\sigma_{3}=$15KPa | | | | |
| --- | --- | --- | --- | --- |
|  | $\sigma_{d}$/(MPa) | | | |
| N | 30 | 55 | 75 | 105 |
| 0 | 321.59 | 313.11 | 296.47 | 282.54 |
| 1 | 295.54 | 271.84 | 258.45 | 247.65 |
| 2 | 274.65 | 249.46 | 231.82 | 221.16 |
| 3 | 258.08 | 232.71 | 206.71 | 190.84 |
| 4 | 246.25 | 207.62 | 183.25 | 157.64 |
| 5 | 227.38 | 194.54 | 167.18 | 142.61 |
| 6 | 222.82 | 190.35 | 163.07 | 136.54 |

Fig 13. Comparison of measured and predicted values.

Model prediction results

| $\sigma_{d}$/(kPa) | $\sigma_{3}$/(kPa) | C/% | N/times | Experimental value /MPa | Predicted value /MPa | MAE | RMSE | RD |
| --- | --- | --- | --- | --- | --- | --- | --- | --- |
| 30 | 15 | 7% | 0 | 255.58 | 245.08 | 13.30 | 17.50 | 5.60% |
| 30 | 30 | 7% | 0 | 273.26 | 279.28 | 13.30 | 17.50 | 5.60% |
| 30 | 45 | 7% | 0 | 286.32 | 302.80 | 13.30 | 17.50 | 5.60% |
| 30 | 60 | 7% | 0 | 300.47 | 320.42 | 13.30 | 17.50 | 5.60% |
| 55 | 15 | 7% | 0 | 242.84 | 223.42 | 13.30 | 17.50 | 5.60% |
| 55 | 30 | 7% | 0 | 266.84 | 249.50 | 13.30 | 17.50 | 5.60% |
| 55 | 45 | 7% | 0 | 281.62 | 269.16 | 13.30 | 17.50 | 5.60% |
| 55 | 60 | 7% | 0 | 292.25 | 284.86 | 13.30 | 17.50 | 5.60% |
| 75 | 15 | 7% | 0 | 229.94 | 215.04 | 13.30 | 17.50 | 5.60% |
| 75 | 30 | 7% | 0 | 251.64 | 236.94 | 13.30 | 17.50 | 5.60% |
| 75 | 45 | 7% | 0 | 264.18 | 254.16 | 13.30 | 17.50 | 5.60% |
| 75 | 60 | 7% | 0 | 280.45 | 268.33 | 13.30 | 17.50 | 5.60% |
| 105 | 15 | 7% | 0 | 203.48 | 207.81 | 13.30 | 17.50 | 5.60% |
| 105 | 30 | 7% | 0 | 225.23 | 225.47 | 13.30 | 17.50 | 5.60% |
| 105 | 45 | 7% | 0 | 242.02 | 239.96 | 13.30 | 17.50 | 5.60% |
| 105 | 60 | 7% | 0 | 258.45 | 252.26 | 13.30 | 17.50 | 5.60% |
| 30 | 15 | 7% | 3 | 194.10 | 188.12 | 13.30 | 17.50 | 5.60% |
| 30 | 30 | 7% | 3 | 220.04 | 214.36 | 13.30 | 17.50 | 5.60% |
| 30 | 45 | 7% | 3 | 235.14 | 232.42 | 13.30 | 17.50 | 5.60% |
| 30 | 60 | 7% | 3 | 252.48 | 245.94 | 13.30 | 17.50 | 5.60% |
| 55 | 15 | 7% | 3 | 175.48 | 171.49 | 13.30 | 17.50 | 5.60% |
| 55 | 30 | 7% | 3 | 197.87 | 191.50 | 13.30 | 17.50 | 5.60% |
| 55 | 45 | 7% | 3 | 211.41 | 206.59 | 13.30 | 17.50 | 5.60% |
| 55 | 60 | 7% | 3 | 229.14 | 218.64 | 13.30 | 17.50 | 5.60% |
| 75 | 15 | 7% | 3 | 153.64 | 165.06 | 13.30 | 17.50 | 5.60% |
| 75 | 30 | 7% | 3 | 178.24 | 181.86 | 13.30 | 17.50 | 5.60% |
| 75 | 45 | 7% | 3 | 196.21 | 195.08 | 13.30 | 17.50 | 5.60% |
| 75 | 60 | 7% | 3 | 213.04 | 205.96 | 13.30 | 17.50 | 5.60% |
| 105 | 15 | 7% | 3 | 134.58 | 159.50 | 13.30 | 17.50 | 5.60% |
| 105 | 30 | 7% | 3 | 156.28 | 173.06 | 13.30 | 17.50 | 5.60% |
| 105 | 45 | 7% | 3 | 180.11 | 184.19 | 13.30 | 17.50 | 5.60% |
| 105 | 60 | 7% | 3 | 194.35 | 193.62 | 13.30 | 17.50 | 5.60% |
| 30 | 15 | 7% | 6 | 174.80 | 169.69 | 13.30 | 17.50 | 5.60% |
| 30 | 30 | 7% | 6 | 195.68 | 193.37 | 13.30 | 17.50 | 5.60% |
| 30 | 45 | 7% | 6 | 211.61 | 209.65 | 13.30 | 17.50 | 5.60% |
| 30 | 60 | 7% | 6 | 230.78 | 221.85 | 13.30 | 17.50 | 5.60% |
| 55 | 15 | 7% | 6 | 152.84 | 154.69 | 13.30 | 17.50 | 5.60% |
| 55 | 30 | 7% | 6 | 176.48 | 172.75 | 13.30 | 17.50 | 5.60% |
| 55 | 45 | 7% | 6 | 198.24 | 186.36 | 13.30 | 17.50 | 5.60% |
| 55 | 60 | 7% | 6 | 215.64 | 197.23 | 13.30 | 17.50 | 5.60% |

Model prediction results

| $\sigma_{d}$/kPa | σ_3_/kPa | C/% | N/times | Experimental value /MPa | Predicted value /MPa | MAE | RMSE | RD |
| --- | --- | --- | --- | --- | --- | --- | --- | --- |
| 75 | 15 | 7% | 6 | 125.65 | 148.89 | 13.30 | 17.50 | 5.60% |
| 75 | 30 | 7% | 6 | 147.48 | 164.05 | 13.30 | 17.50 | 5.60% |
| 75 | 45 | 7% | 6 | 171.89 | 175.98 | 13.30 | 17.50 | 5.60% |
| 75 | 60 | 7% | 6 | 196.35 | 185.79 | 13.30 | 17.50 | 5.60% |
| 105 | 15 | 7% | 6 | 101.25 | 143.88 | 13.30 | 17.50 | 5.60% |
| 105 | 30 | 7% | 6 | 127.32 | 156.11 | 13.30 | 17.50 | 5.60% |
| 105 | 45 | 7% | 6 | 150.14 | 166.15 | 13.30 | 17.50 | 5.60% |
| 105 | 60 | 7% | 6 | 177.15 | 174.66 | 13.30 | 17.50 | 5.60% |
| 30 | 15 | 9% | 0 | 278.43 | 277.99 | 13.30 | 17.50 | 5.60% |
| 30 | 30 | 9% | 0 | 297.85 | 316.77 | 13.30 | 17.50 | 5.60% |
| 30 | 45 | 9% | 0 | 318.96 | 343.45 | 13.30 | 17.50 | 5.60% |
| 30 | 60 | 9% | 0 | 326.61 | 363.43 | 13.30 | 17.50 | 5.60% |
| 55 | 15 | 9% | 0 | 276.59 | 253.41 | 13.30 | 17.50 | 5.60% |
| 55 | 30 | 9% | 0 | 290.86 | 282.99 | 13.30 | 17.50 | 5.60% |
| 55 | 45 | 9% | 0 | 313.72 | 305.29 | 13.30 | 17.50 | 5.60% |
| 55 | 60 | 9% | 0 | 317.68 | 323.10 | 13.30 | 17.50 | 5.60% |
| 75 | 15 | 9% | 0 | 261.90 | 243.91 | 13.30 | 17.50 | 5.60% |
| 75 | 30 | 9% | 0 | 289.39 | 268.74 | 13.30 | 17.50 | 5.60% |
| 75 | 45 | 9% | 0 | 287.69 | 288.28 | 13.30 | 17.50 | 5.60% |
| 75 | 60 | 9% | 0 | 315.23 | 304.35 | 13.30 | 17.50 | 5.60% |
| 105 | 15 | 9% | 0 | 231.76 | 235.71 | 13.30 | 17.50 | 5.60% |
| 105 | 30 | 9% | 0 | 252.71 | 255.74 | 13.30 | 17.50 | 5.60% |
| 105 | 45 | 9% | 0 | 269.13 | 272.18 | 13.30 | 17.50 | 5.60% |
| 105 | 60 | 9% | 0 | 288.69 | 286.13 | 13.30 | 17.50 | 5.60% |
| 30 | 15 | 9% | 3 | 234.00 | 213.37 | 13.30 | 17.50 | 5.60% |
| 30 | 30 | 9% | 3 | 252.39 | 243.14 | 13.30 | 17.50 | 5.60% |
| 30 | 45 | 9% | 3 | 261.24 | 263.62 | 13.30 | 17.50 | 5.60% |
| 30 | 60 | 9% | 3 | 287.57 | 278.96 | 13.30 | 17.50 | 5.60% |
| 55 | 15 | 9% | 3 | 198.12 | 194.51 | 13.30 | 17.50 | 5.60% |
| 55 | 30 | 9% | 3 | 214.89 | 217.21 | 13.30 | 17.50 | 5.60% |
| 55 | 45 | 9% | 3 | 230.01 | 234.33 | 13.30 | 17.50 | 5.60% |
| 55 | 60 | 9% | 3 | 261.45 | 248.00 | 13.30 | 17.50 | 5.60% |
| 75 | 15 | 9% | 3 | 167.16 | 187.22 | 13.30 | 17.50 | 5.60% |
| 75 | 30 | 9% | 3 | 196.24 | 206.28 | 13.30 | 17.50 | 5.60% |
| 75 | 45 | 9% | 3 | 218.77 | 221.27 | 13.30 | 17.50 | 5.60% |
| 75 | 60 | 9% | 3 | 239.46 | 233.61 | 13.30 | 17.50 | 5.60% |
| 105 | 15 | 9% | 3 | 150.33 | 180.92 | 13.30 | 17.50 | 5.60% |
| 105 | 30 | 9% | 3 | 171.13 | 196.29 | 13.30 | 17.50 | 5.60% |
| 105 | 45 | 9% | 3 | 199.38 | 208.91 | 13.30 | 17.50 | 5.60% |
| 105 | 60 | 9% | 3 | 211.45 | 219.62 | 13.30 | 17.50 | 5.60% |
| 30 | 15 | 9% | 6 | 219.56 | 192.47 | 13.30 | 17.50 | 5.60% |

Model prediction results

| $\sigma_{d}$/kPa | σ_3_/kPa | C/% | N/times | Experimental value /MPa | Predicted value /MPa | MAE | RMSE | RD |
| --- | --- | --- | --- | --- | --- | --- | --- | --- |
| 30 | 30 | 9% | 6 | 222.49 | 219.33 | 13.30 | 17.50 | 5.60% |
| 30 | 45 | 9% | 6 | 241.87 | 237.80 | 13.30 | 17.50 | 5.60% |
| 30 | 60 | 9% | 6 | 262.40 | 251.64 | 13.30 | 17.50 | 5.60% |
| 55 | 15 | 9% | 6 | 168.89 | 175.46 | 13.30 | 17.50 | 5.60% |
| 55 | 30 | 9% | 6 | 199.25 | 195.94 | 13.30 | 17.50 | 5.60% |
| 55 | 45 | 9% | 6 | 222.82 | 211.38 | 13.30 | 17.50 | 5.60% |
| 55 | 60 | 9% | 6 | 240.22 | 223.71 | 13.30 | 17.50 | 5.60% |
| 75 | 15 | 9% | 6 | 141.73 | 168.88 | 13.30 | 17.50 | 5.60% |
| 75 | 30 | 9% | 6 | 167.68 | 186.08 | 13.30 | 17.50 | 5.60% |
| 75 | 45 | 9% | 6 | 187.19 | 199.60 | 13.30 | 17.50 | 5.60% |
| 75 | 60 | 9% | 6 | 225.41 | 210.73 | 13.30 | 17.50 | 5.60% |
| 105 | 15 | 9% | 6 | 112.08 | 163.20 | 13.30 | 17.50 | 5.60% |
| 105 | 30 | 9% | 6 | 142.22 | 177.07 | 13.30 | 17.50 | 5.60% |
| 105 | 45 | 9% | 6 | 171.76 | 188.45 | 13.30 | 17.50 | 5.60% |
| 105 | 60 | 9% | 6 | 198.05 | 198.11 | 13.30 | 17.50 | 5.60% |
| 30 | 15 | 11% | 0 | 305.44 | 314.58 | 13.30 | 17.50 | 5.60% |
| 30 | 30 | 11% | 0 | 324.66 | 358.48 | 13.30 | 17.50 | 5.60% |
| 30 | 45 | 11% | 0 | 341.52 | 388.67 | 13.30 | 17.50 | 5.60% |
| 30 | 60 | 11% | 0 | 355.03 | 411.28 | 13.30 | 17.50 | 5.60% |
| 55 | 15 | 11% | 0 | 313.11 | 286.77 | 13.30 | 17.50 | 5.60% |
| 55 | 30 | 11% | 0 | 334.48 | 320.25 | 13.30 | 17.50 | 5.60% |
| 55 | 45 | 11% | 0 | 341.65 | 345.49 | 13.30 | 17.50 | 5.60% |
| 55 | 60 | 11% | 0 | 357.07 | 365.64 | 13.30 | 17.50 | 5.60% |
| 75 | 15 | 11% | 0 | 296.47 | 276.02 | 13.30 | 17.50 | 5.60% |
| 75 | 30 | 11% | 0 | 324.69 | 304.13 | 13.30 | 17.50 | 5.60% |
| 75 | 45 | 11% | 0 | 337.85 | 326.23 | 13.30 | 17.50 | 5.60% |
| 75 | 60 | 11% | 0 | 352.11 | 344.42 | 13.30 | 17.50 | 5.60% |
| 105 | 15 | 11% | 0 | 282.54 | 266.74 | 13.30 | 17.50 | 5.60% |
| 105 | 30 | 11% | 0 | 294.36 | 289.40 | 13.30 | 17.50 | 5.60% |
| 105 | 45 | 11% | 0 | 311.69 | 308.01 | 13.30 | 17.50 | 5.60% |
| 105 | 60 | 11% | 0 | 327.99 | 323.80 | 13.30 | 17.50 | 5.60% |
| 30 | 15 | 11% | 3 | 260.08 | 241.46 | 13.30 | 17.50 | 5.60% |
| 30 | 30 | 11% | 3 | 289.49 | 275.15 | 13.30 | 17.50 | 5.60% |
| 30 | 45 | 11% | 3 | 290.24 | 298.33 | 13.30 | 17.50 | 5.60% |
| 30 | 60 | 11% | 3 | 327.55 | 315.68 | 13.30 | 17.50 | 5.60% |
| 55 | 15 | 11% | 3 | 232.71 | 220.12 | 13.30 | 17.50 | 5.60% |
| 55 | 30 | 11% | 3 | 242.80 | 245.81 | 13.30 | 17.50 | 5.60% |
| 55 | 45 | 11% | 3 | 260.37 | 265.18 | 13.30 | 17.50 | 5.60% |
| 55 | 60 | 11% | 3 | 310.36 | 280.65 | 13.30 | 17.50 | 5.60% |
| 75 | 15 | 11% | 3 | 206.71 | 211.87 | 13.30 | 17.50 | 5.60% |
| 75 | 30 | 11% | 3 | 245.57 | 233.44 | 13.30 | 17.50 | 5.60% |

Model prediction results

| $\sigma_{d}$/kPa | σ_3_/kPa | C/% | N/times | Experimental value /MPa | Predicted value /MPa | MAE | RMSE | RD |
| --- | --- | --- | --- | --- | --- | --- | --- | --- |
| 75 | 45 | 11% | 3 | 277.24 | 250.40 | 13.30 | 17.50 | 5.60% |
| 75 | 60 | 11% | 3 | 295.84 | 264.37 | 13.30 | 17.50 | 5.60% |
| 105 | 15 | 11% | 3 | 190.84 | 204.74 | 13.30 | 17.50 | 5.60% |
| 105 | 30 | 11% | 3 | 222.24 | 222.13 | 13.30 | 17.50 | 5.60% |
| 105 | 45 | 11% | 3 | 250.86 | 236.42 | 13.30 | 17.50 | 5.60% |
| 105 | 60 | 11% | 3 | 275.48 | 248.53 | 13.30 | 17.50 | 5.60% |
| 30 | 15 | 11% | 3 | 222.82 | 217.81 | 13.30 | 17.50 | 5.60% |
| 30 | 30 | 11% | 3 | 252.97 | 248.20 | 13.30 | 17.50 | 5.60% |
| 30 | 45 | 11% | 3 | 276.46 | 269.11 | 13.30 | 17.50 | 5.60% |
| 30 | 60 | 11% | 3 | 298.35 | 284.77 | 13.30 | 17.50 | 5.60% |
| 55 | 15 | 11% | 3 | 190.35 | 198.56 | 13.30 | 17.50 | 5.60% |
| 55 | 30 | 11% | 3 | 229.45 | 221.74 | 13.30 | 17.50 | 5.60% |
| 55 | 45 | 11% | 3 | 255.46 | 239.21 | 13.30 | 17.50 | 5.60% |
| 55 | 60 | 11% | 3 | 272.96 | 253.16 | 13.30 | 17.50 | 5.60% |
| 75 | 15 | 11% | 3 | 163.07 | 191.12 | 13.30 | 17.50 | 5.60% |
| 75 | 30 | 11% | 3 | 194.47 | 210.57 | 13.30 | 17.50 | 5.60% |
| 75 | 45 | 11% | 3 | 207.92 | 225.88 | 13.30 | 17.50 | 5.60% |
| 75 | 60 | 11% | 3 | 263.95 | 238.47 | 13.30 | 17.50 | 5.60% |
| 105 | 15 | 11% | 3 | 126.56 | 184.69 | 13.30 | 17.50 | 5.60% |
| 105 | 30 | 11% | 3 | 162.03 | 200.38 | 13.30 | 17.50 | 5.60% |
| 105 | 45 | 11% | 3 | 200.42 | 213.26 | 13.30 | 17.50 | 5.60% |
| 105 | 60 | 11% | 3 | 225.85 | 224.19 | 13.30 | 17.50 | 5.60% |

|  |  |  |
| --- | --- | --- |
| N=0 | N=4 | N=6 |

**Fig 14. SEM pattern under different N**.

|  |  |  |
| --- | --- | --- |
| N=0 | N=4 | N=6 |

**Fig 15. XRD pattern under different N.**
